# Improving upon the efficiency of complete case analysis when covariates are MNAR

**DOI:** 10.1093/biostatistics/kxu023

**Published:** 2014-06-06

**Authors:** Jonathan W. Bartlett, James R. Carpenter, Kate Tilling, Stijn Vansteelandt

**Affiliations:** Centre for Statistical Methodology, London School of Hygiene and Tropical Medicine, Keppel Street, London WC1E 7HT, UK; Centre for Statistical Methodology, London School of Hygiene and Tropical Medicine, Keppel Street, London WC1E 7HT, UK and MRC Clinical Trial Trials Unit, Kingsway, London WC2B 6NH, UK; School of Social and Community Medicine, University of Bristol, Canynge Hall, 39 Whatley Road, Bristol BS8 2PS, UK; Department of Applied Mathematics, Computer Science and Statistics, Ghent University, Krijgslaan, 281 S9, B-9000 Ghent, Belgium

**Keywords:** Complete case analysis, Missing covariates, Missing not at random, Multiple imputation

## Abstract

Missing values in covariates of regression models are a pervasive problem in empirical research. Popular approaches for analyzing partially observed datasets include complete case analysis (CCA), multiple imputation (MI), and inverse probability weighting (IPW). In the case of missing covariate values, these methods (as typically implemented) are valid under different missingness assumptions. In particular, CCA is valid under missing not at random (MNAR) mechanisms in which missingness in a covariate depends on the value of that covariate, but is conditionally independent of outcome. In this paper, we argue that in some settings such an assumption is more plausible than the missing at random assumption underpinning most implementations of MI and IPW. When the former assumption holds, although CCA gives consistent estimates, it does not make use of all observed information. We therefore propose an augmented CCA approach which makes the same conditional independence assumption for missingness as CCA, but which improves efficiency through specification of an additional model for the probability of missingness, given the fully observed variables. The new method is evaluated using simulations and illustrated through application to data on reported alcohol consumption and blood pressure from the US National Health and Nutrition Examination Survey, in which data are likely MNAR independent of outcome.

## 1. Introduction

Missing data in covariates of regression models are a common problem in epidemiological and clinical studies. Three commonly applied approaches for analyzing datasets with missing covariates are complete case analysis (CCA), multiple imputation (MI), and inverse probability weighting (IPW). As typically implemented, each make different assumptions about various aspects of either the data or the mechanism causing missingness.

CCA has the advantage of being simple to apply, and is usually the default method in statistical packages. It is well known that CCA gives valid inferences when data are missing completely at random. Perhaps less widely appreciated is the fact that CCA gives valid inferences provided that the probability of being a complete case is independent of the outcome in the model of interest, conditional on the model's covariates (Little and Rubin, 2002). In particular, CCA is valid under missing not at random (MNAR) mechanisms in which missingness in a covariate is dependent on the value of that covariate, but is conditionally independent of outcome (White and Carlin, 2010). However, even when this assumption holds, it does not make full use of the observed information, since observed data from the incomplete cases are discarded.

MI involves creating multiple imputed values for each missing value, creating a number of imputed datasets. Each imputed dataset is analyzed separately, and their estimates combined using rules developed by Rubin (1987). If data are missing at random (MAR) and the imputation model is correctly specified, MI gives valid inferences, and is generally more efficient than CCA since it uses the observed data from incomplete cases and potentially also from auxiliary variables which are not involved in the model of interest. This had led to MI being widely advocated and used in applications (Sterne *and others*, 2009).

IPW is also typically implemented assuming MAR. IPW avoids the necessity to model the distribution of the partially observed variables, and instead relies on a model for the missingness mechanism (Seaman and White, 2011). However, IPW is difficult to implement with non-monotone missingness, and is usually less efficient than MI. In recent years, doubly robust MAR estimators have been proposed which attempt to improve upon the efficiency of IPW, and which also give additional robustness to model mis-specification (Carpenter *and others*, 2006; Tsiatis, 2006).

In this article, we argue that in some settings an MNAR missingness mechanism under which CCA is valid is more plausible than an MAR mechanism which is required for validity of a conventional MI or IPW analysis. In such settings, it may therefore be preferable from the perspective of bias to use CCA. However, as previously noted, CCA is inefficient because it fails to draw on the information available in those subjects with some data missing. To address this, we develop an augmented CCA estimation method which can improve upon the efficiency of CCA, through specification of an additional model for the probability of missingness given the fully observed variables.

In Section 2, we argue why the CCA assumption may in some settings be more plausible than MAR, and propose an estimation method which makes this assumption but which draws on the information available from incomplete cases. We explore the performance of the proposed method in simulations in Section 3. In Section 4, we illustrate the method using alcohol and blood pressure data from the US National Health and Nutrition Examination Survey (NHANES), and give some concluding comments in Section 5.

## 2. Improving upon the efficiency of CCA

Consider a study where an outcome $Y$ and covariates $X$ and $Z$ are intended to be collected for a random sample of independent subjects. Either of $X$ and $Z$ (or both) may be vector valued. We assume throughout that the following conditional mean model holds:

(2.1) \begin{equation*}\label{eq2.1} E(Y|X,Z) = g(X,Z;\beta), \end{equation*}

where the function $g(X,Z; \beta)$ is a known smooth function of $\beta $, a finite-dimensional parameter to be estimated, with true value $\beta ^{*}$. Conditional mean models include familiar models such as linear and logistic regression.

### 2.1 Missingness assumptions

We assume that $Y$ and $Z$ are fully observed, while the covariate $X$ is partially observed. In the case where $X$ is vector valued, we assume that for a given subject either all elements of $X$ are observed or all elements are missing (see Section 5 for discussion on extensions to more complex missingness patterns). We let $R$ denote whether $X$ is observed ($R=1$) or missing ($R=0$). Within this setup, the MAR assumption, upon which most MI and IPW methods rely, is that $R \perp \!\!\!\perp X | Y,Z$. In contrast, CCA provides consistent parameter estimates and valid inferences if missingness is independent of outcome conditional on covariates, i.e. $R \perp \!\!\!\perp Y | X,Z$. This condition encompasses both certain MAR mechanisms, whereby $R$ depends on the fully observed covariates $Z$, but given this is independent of $X$ and $Y$, and certain MNAR mechanisms, whereby $R$ depends on $X$ (and possibly $Z$), but given this, is independent of $Y$. Unfortunately, as discussed by White and Carlin (2010), it is usually impossible to distinguish on the basis of the observed data which, if either, missingness assumption is appropriate. In Appendix A of supplementary material available at *Biostatistics* online, we show that there exist exceptions whereby the assumption that $R \perp \!\!\!\perp Y | X,Z$ may be testable on the basis of the observed data. However, generally our contextual knowledge must guide us as to which, if either, is plausible.

For some questions (and variables), it may be deemed likely from contextual knowledge and experience that propensity to respond to the question is at least partly determined by the value of that variable, such that missingness is not at random. Examples include surveys in which participants are asked about their income, with those with low or high income generally considered less likely to respond (Little and Zhang, 2011). In Section 4, we consider data on alcohol consumption and blood pressure from NHANES, in which we argue missingness in alcohol consumption is likely to depend largely on alcohol consumption itself, and given consumption (and other covariates), be independent of blood pressure level. As we describe in further detail in Appendix B of supplementary material available at *Biostatistics* online, in other settings the assumption that $R \perp \!\!\!\perp Y | X,Z$ may be plausible if the covariate $X$ is measured much earlier in time than the outcome $Y$. Thus, sometimes the assumption that $R \perp \!\!\!\perp Y | X,Z$ may be plausible, while the MAR assumption that $R \perp \!\!\!\perp X | Y,Z$ will not be.

### 2.2 Estimation with full data

Before considering estimation with partially observed $X$, we first consider estimation in the absence of missing data. Let $(X_{i},Z_{i},Y_{i},R_{i})$, $i=1,\ldots ,n$ denote an i.i.d. sample of $n$ subjects. All regular and asymptotically linear estimators of the parameter $\beta $ indexing the conditional mean model of interest (equation (2.1)) can be expressed (up to asymptotic equivalence) as the solution $\hat {\beta }$ to an estimating equation of the form

(2.2) \begin{equation*}\label{eq2.2} \sum^{n}_{i=1} d(X_{i},Z_{i}) \epsilon_{i}(\hat{\beta})=0, \end{equation*}

where $d(X,Z)$ is a vector-valued function of $(X,Z)$ with dimension that of $\beta $, and $\epsilon (\beta)=Y-g(X,Z;\beta)$ (Rotnitzky and Robins, 1997). That estimating $\beta $ by solving such an estimating equation results in a consistent estimator follows from the fact that the expectation of the estimating function $d(X,Z)\epsilon (\beta)$ is zero when evaluated at the true value $\beta ^{*}$. The efficiency of the estimator depends on the choice of the function $d(X,Z)$, with the optimal choice being given by $ d(X,Z) = (\partial g(X,Z;\beta ^{*})/\partial \beta) {\mathrm {Var}}(Y|X,Z)^{-1}$.

### 2.3 Estimation with partially observed $X$

Now suppose that $X$ is partially observed, with $R \perp \!\!\!\perp Y | X,Z$. As described previously, under this assumption CCA gives valid inferences, but fails to draw on the observed information in the incomplete cases. In Appendix C of supplementary material available at *Biostatistics* online, we show that if a fully parametric model $f(Y|X,Z,\beta)$ is assumed (rather than the conditional mean model of (2.1)), without making further assumptions all regular and asymptotically linear estimators of $\beta $ only use information from the complete cases. It follows that we must make additional assumptions in order to extract information from the incomplete cases.

One route to gaining efficiency over CCA is to take a fully parametric approach, which involves specifying parametric models for $P(R|X,Z)$, for $f(Y|X,Z)$, and for $f(X|Z)$, or a semi-parametric approach as in Rotnitzky and Robins (1997), based only (in additional to the conditional mean model of interest) on a parametric model for $P(R|X,Z)$. These are somewhat unappealing because the validity of resulting inferences will depend on the correct specification of these models. In particular, since a model for $P(R|X,Z)$ cannot be directly estimated using the observed data (whenever $R=0$, $X$ is missing), ensuring that this model is correctly specified would be difficult.

Instead, we consider estimation of $\beta $ given specification of a model $P(R|Y,Z;\alpha)$, indexed by parameter $\alpha $, for $P(R|Y,Z)$. Note that, under our assumptions, this is not a model for the underlying missingness mechanism $P(R|X,Z)$. However, since the model $P(R|Y,Z;\alpha)$ only involves fully observed variables (unlike a model for the underlying missingness mechanism), estimation of $\alpha $ is standard, e.g. by maximum likelihood (ML). Specifically, we assume that the following logistic model holds:

(2.3) \begin{equation*}\label{eq2.3} P(R=1|Y,Z;\alpha)= \pi(Y,Z;\alpha) =\hbox{expit}(h(Y,Z;\alpha)), \end{equation*}

where $h(Y,Z;\alpha)$ is a known function, linear in $\alpha $, and $\alpha $ is a finite-dimensional parameter with true value $\alpha ^{*}$.

Suppose for the moment that the true value of $\alpha $, $\alpha ^{*}$ is known. Letting $O_{i}=(Y_{i},Z_{i},R_{i},R_{i}X_{i})^{T}$ denote the data observed from subject $i$, it is then easily verified that the (infeasible) estimator $\hat {\beta }_{{\mathrm {INF}}}$ which solves the estimating equation

(2.4) \begin{equation*}\label{eq2.4} \sum^{n}_{i=1} m(O_{i},\alpha^{\ast},\hat{\beta}_{{\rm INF}}) =0, \end{equation*}

where

(2.5) \begin{equation*}\label{eq2.5} m(O,\alpha,\beta) = R d(X,Z) \epsilon(\beta) + \{R - \pi(Y,Z;\alpha)\} \phi(Y,Z,\beta), \end{equation*}

and where $d(X,Z)$ and $\phi (Y,Z,\beta)$ are arbitrary functions with dimension the same as $\beta $, is consistent and asymptotically normal. The first part is identical to the CCA estimating function, which has mean zero (at $\beta ^{*}$) when $R \perp \!\!\!\perp Y | X,Z$. The second part, to which both subjects with $X$ observed and those with $X$ missing contribute, has mean zero provided that the model for $P(R|Y,Z)$ is correctly specified, since then $E(R - \pi (Y,Z;\alpha ^{*})|Y,Z)=0$.

In practice, $\alpha $ must be estimated. We assume that $\alpha $ is estimated by its MLE, which is the value $\hat {\alpha }$ solving the likelihood score equations

\[ \sum^{n}_{i=1} \{R_{i} - \pi(Y_i,Z_i;\hat{\alpha})\}h_{\alpha}(Y_i,Z_i)=0,\%\label{alphaestimatingequation} \]

with $h_{\alpha }(Y,Z)= ({\partial }/{\partial \alpha }) h(Y,Z;\alpha)$. The parameter $\beta $ of interest can then be estimated by solving the estimating equation (2.4), replacing the unknown $\alpha ^{*}$ by its MLE $\hat {\alpha }$, i.e. by solving

(2.6) \begin{equation*}\label{eq2.6} \sum^{n}_{i=1} m(O_{i},\hat{\alpha},\hat{\beta}_{{\mathrm{ACC}}}) =0. \end{equation*}

In Appendix D.1 of supplementary material available at *Biostatistics* online, we show that under suitable regularity conditions, this augmented complete case (ACC) estimator $\hat {\beta }_{{\rm ACC}}$ is consistent and asymptotically normal, with influence function

(2.7) \begin{equation*}\label{eq2.7} -G^{-1}_{\beta} [Rd(X,Z)\epsilon(\beta^{*}) +\{R-\pi(Y,Z;\alpha^{*})\} \tilde{\phi}(Y,Z,\alpha^{*},\beta^{*})], \end{equation*}

where

(2.8) \begin{equation*}\label{eq2.8} G_{\beta} = E\left\{\frac{\partial}{\partial \beta^{T}} R d(X,Z) \epsilon(\beta^{*}) \right\} \end{equation*}

and

(2.9) \begin{align} \tilde{\phi}(y,z,\alpha,\beta)&=\phi(y,z,\beta)-E[\pi(Y,Z;\alpha)\{1-\pi(Y,Z;\alpha)\}\phi(Y,Z,\beta)h^{T}_{\alpha}(Y,Z)] \nonumber \\ &\quad \times \{ E[\pi(Y,Z;\alpha)\{1-\pi(Y,Z;\alpha)\}h_{\alpha}(Y,Z)h^{T}_{\alpha}(Y,Z)]\}^{-1} h_{\alpha}(y,z). \label{eq2.9} \end{align}

The asymptotic variance of $\hat {\beta }_{{\mathrm {ACC}}}$ is equal to $n^{-1}$ times the variance of the influence function (as given by (2.7)), and this can be estimated using (2.8) and (2.9), replacing expectations and variances by their empirical counterparts, and $\alpha ^{*}$ and $\beta ^{*}$ by their corresponding sample estimates.

The choices of the functions $d(X,Z)$ and $\phi (Y,Z,\beta)$ affect the efficiency of $\hat {\beta }_{{\mathrm {ACC}}}$. For simplicity, we consider how to choose $\phi (Y,Z,\beta)$ in order to minimize the variance of $\hat {\beta }_{{\mathrm {ACC}}}$ for a given choice of $d(X,Z)$ (e.g. the choice we would use with full data). In Appendix D.2 of supplementary material available at *Biostatistics* online, we show that the optimal function $\phi ^{{\rm opt}}(Y,Z,\beta)$ is given by

(2.10) \begin{equation*}\label{eq2.10} \phi^{{\mathrm{opt}}}(Y,Z,\beta) = - E[ d(X,Z)\epsilon(\beta) |Y,Z,R=1], \end{equation*}

which in particular improves upon the efficiency of CCA, which is obtained by choosing $\phi (Y,Z,\beta)=0$. We let $\hat {\beta }_{{\mathrm {ACC\hbox {-}}TRUE}}$ denote the estimator which uses $\phi (Y,Z,\beta)=\phi ^{{\mathrm {opt}}}(Y,Z,\beta)$.

### 2.4 Implementation

The optimal choice $\phi ^{{\mathrm {opt}}}(Y,Z,\beta)$ depends on aspects of the data generating mechanism about which we have not made assumptions. We consider two approaches for estimating $\phi ^{{\mathrm {opt}}}(Y,Z,\beta)$.

The first is to posit a parametric working model $f(X|R=1,Y,Z;\eta)$ and calculate the expectations required in $\phi ^{{\mathrm {opt}}}(Y,Z,\beta)$. We denote the resulting estimator by $\hat {\beta }_{{\mathrm {ACC\hbox {-}}WM}}$. Note that while mis-specification of this working model will affect the efficiency of the estimator, it will not affect its consistency. This also means that, by Newey and McFadden (1994, Theorem 6.2), estimation of $\eta $ can be ignored when calculating variance estimates, and that the estimator in which $\eta $ is estimated will have the same asymptotic efficiency as the estimator which uses the probability limit value $\eta ^{*}$. If the working model is correctly specified, $\hat {\beta }_{{\mathrm {ACC\hbox {-}}WM}}$ will thus have the same efficiency as $\hat {\beta }_{{\rm ACC\hbox {-}TRUE}}$. In our simulations and illustrative example, we estimate $\eta $ by ML in the complete cases and calculate the expectations involved in $\phi ^{{\rm opt}}(Y,Z,\beta)$ by Monte-Carlo integration. This involves generating $m$ improper imputations from the implied distribution $f(X|R=1,Y,Z;\hat {\eta })$ for each subject, and approximating the required expectations by their empirical means based on these imputations. Inferences may be anti-conservative if a small value of $m$ is used, although we did not find this to be the case in simulations (see Section 3).

If the posited working model $f(X|R=1,Y,Z;\eta)$ is mis-specified, there is no guarantee that $\hat {\beta }_{{\mathrm {ACC\hbox {-}}WM}}$ will improve upon the efficiency of CCA. In Appendix D.3 of supplementary material available at *Biostatistics* online, we give details of a modified estimator $\hat {\beta }_{{\rm ACC2}}$ which, for a given choice of $\phi (Y,Z,\beta)$ (or working model used to estimate $\phi ^{{\rm opt}}(Y,Z,\beta)$), ensures that estimates are at least as efficient as CCA. We denote the corresponding estimator which uses a parametric working model to estimate $\phi ^{{\mathrm {opt}}}(Y,Z,\beta)$ by $\hat {\beta }_{{\rm ACC2\hbox {-}WM}}$.

The second approach we consider is non-parametric estimation of $\phi ^{{\mathrm {opt}}}(Y,Z,\beta)$ using kernel regression. Following similar approaches used in the MAR context (Qi *and others*, 2005), we estimate $\phi ^{{\rm opt}}(Y,Z,\beta)$ using the Nadaraya–Watson estimator. Letting $K$ denote a kernel function, this is given by

\[ \hat{\phi}^{{\rm opt}}(Y,Z,\beta) = \frac{\sum^{n}_{i=1} R_{i} K_{h}((Y,Z)-(Y_{i},Z_{i})) \{-d(X_{i},Z_{i}) \epsilon_{i}(\beta)\}}{\sum^{n}_{i=1} R_{i}K_{h}((Y,Z)-(Y_{i},Z_{i}))}, \]

where $K_{h}(\cdot)=K(\cdot /h)$ and $h$ denotes a vector of bandwidths. To avoid having to calculate $\hat {\phi }^{{\rm opt}}(Y,Z,\beta)$ repeatedly when solving the estimating equations, one can instead use $\hat {\phi }^{{\rm opt}}(Y,Z,\hat {\beta }_{\mathrm {CCA}})$, where $\hat {\beta }_{\rm CCA}$ denotes the CCA estimator. In Appendix E of supplementary material available at *Biostatistics* online, we show that under suitable regularity conditions, the resulting estimator, denoted by $\hat {\beta }_{{\rm ACC\hbox {-}NP}}$, has the same asymptotic distribution as $\hat {\beta }_{{\mathrm {ACC\hbox {-}}TRUE}}$. This means in particular that, as in the case of a parametric working model, kernel estimation of $\phi ^{{\mathrm {opt}}}(Y,Z,\beta)$ can be ignored for the purposes of variance estimation. Letting $r$ denote the number of continuous components in $(Y,Z)$, the bandwidth conditions of Appendix E of supplementary material available at *Biostatistics* online can be satisfied by choosing $h$ to be of order $n^{-1/p}$, for some integer $p$ with $p>r+4$ and $p>2r$.

In the special case of a linear conditional mean model, $\phi ^{{\mathrm {opt}}}(Y,Z,\beta)$ depends only on $E(X|Y,Z,R=1)$ and $E(X^{2}|Y,Z,R=1)$, and so in the simulation study and illustrative analysis we also implement an estimator $\hat {\beta }_{{\mathrm {ACC\hbox {-}}NP2}}$ in which $\phi ^{{\mathrm {opt}}}(Y,Z,\beta)$ is estimated using the Nadaraya–Watson estimates of $E(X|Y,Z,R=1)$ and $E(X^{2}|Y,Z,R=1)$.

## 3. Simulations

In this section, we present simulation results to examine the performance of the proposed estimator for a linear conditional mean model, and compare it to CCA, MI (assuming MAR), and an IPW CCA (assuming MAR) estimator. The simulation setup is described in detail in Appendix F of supplementary material available at *Biostatistics* online. In brief, for 1000 datasets of size $n=1000$, the observation indicator $R$ was simulated with $P(R=1)=0.5$ and covariates $(Y,X,Z)$ were then generated from a trivariate normal distribution conditional on $R$, such that $R \perp \!\!\!\perp Y | X,Z$. The setup meant that $R|Y,X,Z$ was a logistic regression with $X$ and $Z$ as covariates and that $R|Y,Z$ was a logistic regression with $Y$ and $Z$ as linear covariates. The conditional mean model of interest was

\[ Y|X,Z \sim N(\beta_{0} + \beta_{X} X + \beta_{Z} Z,\sigma^{2}_{\epsilon}), \]

with $\beta _{0}=0$, $\beta _{X}=\beta _{Z}=0.2$, and the coefficient of determination $\approx 0.1$. We present results for the following estimators:

1. CCA;
2. MI, assuming MAR: estimates based on 10 (proper) MIs of $X$, assuming a normal linear regression imputation model for $X|Y,Z;$
3. IPW MAR: the standard IPW CCA estimator assuming MAR, using weights found from a logistic regression model with $Y$ and $Z$ included as linear covariates;
4. ACC estimator, assuming a logistic regression model for $R|Y,Z$:
  - $\hat {\beta }_{{\mathrm {ACC\hbox {-}}TRUE}}$ using the true $\phi ^{{\mathrm {opt}}}(Y,Z,\beta);$
  - $\hat {\beta }_{{\mathrm {ACC\hbox {-}}WM1}}$ with $\phi ^{{\mathrm {opt}}}(Y,Z,\beta)$ estimated using Monte-Carlo integration (10 imputations) based on a parametric working model (normal linear regression for $X|Y,Z,R=1$, with $Y$ and $Z$ as covariates);
  - $\hat {\beta }_{{\mathrm {ACC2\hbox {-}}WM1}}$, using the working model of $\hat {\beta }_{{\mathrm {ACC\hbox {-}}WM1}}$;
  - $\hat {\beta }_{{\mathrm {ACC\hbox {-}}WM2}}$ with $\phi ^{{\mathrm {opt}}}(Y,Z,\beta)$ estimated using Monte-Carlo integration but with a mis-specified working model (normal linear regression with $Y^2$ and $Z^2$ as covariates);
  - $\hat {\beta }_{{\mathrm {ACC2\hbox {-}}WM2}}$ using the working model of $\hat {\beta }_{{\mathrm {ACC\hbox {-}}WM2}}$;
  - $\hat {\beta }_{{\mathrm {ACC\hbox {-}}NP}}$ and $\hat {\beta }_{{\mathrm {ACC\hbox {-}}NP2}}$, using a normal kernel, assuming independence, and with $h_{j}=\hat {\sigma }_{j}n_{\mathrm {CC}}^{-1/7}$ where $\hat {\sigma }_{j}$ denotes the sample standard deviation of the $j$th conditioning variable in the subset where $R=1$ and $n_{\mathrm {CC}}$ denotes the number of complete cases.

Table 1 shows the simulation results, based on 1000 simulations for each scenario. CCA was unbiased for all scenarios, as expected. Both MI and IPW assuming MAR were biased for $\beta _{0}$, but had little bias for $\beta _{X}$ and $\beta _{Z}$. These findings are consistent with the analytical results of Appendix G of supplementary material available at *Biostatistics* online, where we derive analytical expressions for the bias of MI assuming MAR for a simpler parametric linear regression model setting without $Z$.

**Table 1.** Mean $($SD$)$ of estimates over $1000$ simulations

Estimator	$\beta _{0}=0$	$\beta _{X}=1$	$\beta _{Z}=1$
CCA	$-0.003$ (0.062)	0.200 (0.044)	0.199 (0.044)
MI	$-0.099$ (0.052)	0.202 (0.044)	0.200 (0.034)
IPW	$-0.100$ (0.054)	0.202 (0.045)	0.198 (0.044)
$\hat {\beta }_{{\mathrm {ACC\hbox {-}}TRUE}}$	$-$0.003 (0.061)	0.200 (0.043)	0.200 (0.034)
$\hat {\beta }_{{\mathrm {ACC\hbox {-}}WM1}}$	$-$0.003 (0.062)	0.201 (0.045)	0.200 (0.034)
$\hat {\beta }_{{\mathrm {ACC2\hbox {-}}WM1}}$	$-$0.001 (0.062)	0.200 (0.044)	0.200 (0.034)
$\hat {\beta }_{{\mathrm {ACC\hbox {-}}WM2}}$	$-$0.001 (0.064)	0.198 (0.047)	0.201 (0.034)
$\hat {\beta }_{{\mathrm {ACC2\hbox {-}}WM2}}$	$-$0.001 (0.062)	0.199 (0.044)	0.200 (0.034)
$\hat {\beta }_{{\mathrm {ACC\hbox {-}}NP}}$	$-$0.006 (0.064)	0.202 (0.047)	0.195 (0.058)
$\hat {\beta }_{{\mathrm {ACC\hbox {-}}NP2}}$	$-$0.001 (0.061)	0.199 (0.043)	0.201 (0.034)

Estimates from CCA, MI assuming MAR, IPW CCA assuming MAR, and ACC analysis for various choices of $\phi (Y,Z,\beta)$ (see text for details).

The ACC estimator was unbiased for all choices of $\phi (Y,Z,\beta)$, as expected from the asymptotic theory. Using the true optimal $\phi ^{{\mathrm {opt}}}(Y,Z,\beta)$ ($\hat {\beta }_{{\mathrm {ACC\hbox {-}}TRUE}}$) resulted in efficiency gain for $\beta _{Z}$ compared to CCA, but estimates of $\beta _{0}$ and $\beta _{X}$ had similar efficiency to CCA. Using a correctly specified working model ($\hat {\beta }_{{\mathrm {ACC\hbox {-}}WM1}}$) resulted in identical efficiency to $\hat {\beta }_{{\rm ACC\hbox {-}TRUE}}$, in agreement with the asymptotic theory, which states that in our setting there is no cost (asymptotically) to estimating the working model parameters. Since the working model was correct here, as expected the estimator $\hat {\beta }_{{\rm ACC2\hbox {-}WM1}}$ had identical efficiency to $\hat {\beta }_{{\mathrm {ACC\hbox {-}}WM1}}$.

Using a mis-specified working model ($\hat {\beta }_{{\rm ACC\hbox {-}WM2}}$) led to estimates of $\beta _{0}$ and $\beta _{X}$ which were less efficient than CCA, although as predicted from theory estimates remained unbiased. With this mis-specified working model, as predicted, use of $\hat {\beta }_{{\mathrm {ACC2}}}$ ensured that efficiency was at least as good as CCA (in fact $\hat {\beta }_{{\rm ACC2\hbox {-}WM2}}$ had the same efficiency as the optimal estimator).

The non-parametric estimator $\hat {\beta }_{{\rm ACC\hbox {-}NP}}$ was less efficient, with estimates in fact more variable than CCA. However, the estimator $\hat {\beta }_{{\mathrm {ACC\hbox {-}}NP2}}$, which estimated $\phi ^{{\mathrm {opt}}}(Y,Z,\beta)$ using non-parametric estimates of $E(X|Y,Z,R=1)$ and $E(X^{2}|Y,Z,R=1)$, attained the same efficiency as $\hat {\beta }_{{\rm ACC\hbox {-}TRUE}}$.

Table 2 shows the empirical coverage of the nominal 95% confidence intervals for the various ACC estimators, found using the sandwich estimator described in Section 2.3. Coverage was close to the nominal 95% level for all choices of $\phi (Y,Z,\beta)$.

**Table 2.** Coverage of $95\%$ confidence intervals for ACC analyses$,$ from $1000$ simulations

Estimator	$\beta _{0}$	$\beta _{X}$	$\beta _{Z}$
$\hat {\beta }_{{\mathrm {ACC\hbox {-}}TRUE}}$	94.2	94.8	94.9
$\hat {\beta }_{{\mathrm {ACC\hbox {-}}WM1}}$	94.2	93.5	94.8
$\hat {\beta }_{{\mathrm {ACC2\hbox {-}}WM1}}$	93.9	94.1	93.8
$\hat {\beta }_{{\mathrm {ACC\hbox {-}}WM2}}$	93.4	95.7	95.1
$\hat {\beta }_{{\mathrm {ACC2\hbox {-}}WM2}}$	94.0	94.5	94.8
$\hat {\beta }_{{\mathrm {ACC\hbox {-}}NP}}$	95.3	95.4	95.2
$\hat {\beta }_{{\mathrm {ACC\hbox {-}}NP2}}$	93.5	93.0	94.6

## 4. Application to nhanes

To illustrate the proposed method, we consider data on alcohol consumption and systolic blood pressure (SBP) from the 2003–2004 NHANES. We focus on the dependence of SBP on the reported average number of alcoholic drinks consumed per day on days where the participant drank alcohol (obtained via a questionnaire) (“no. drinks”), with adjustment for age and body mass index (BMI). Data are available for $n=2418$ men, for whom $278$ are missing SBP and $181$ are missing BMI. As argued by Little and Zhang (2011), it is plausible that missingness in SBP and BMI is completely at random due to missed visits, and therefore excluding these participants ought not to introduce bias. Amongst the remaining $2111$ participants, $720$ (34.1%) have the alcohol variable missing. It is *a priori* plausible that missingness in the alcohol variable is primarily dependent on the value of the alcohol variable (i.e. MNAR), and given this, and age and BMI, is independent of SBP. Consequently, CCA is expected to give valid inferences, while the MAR assumption likely does not hold.

A logistic regression model was fitted relating whether the alcohol variable was observed, with age, BMI, and SBP (linear and quadratic terms) as covariates (Table 3). There was strong evidence that age was associated with missingness, with increasing age associated with reduced odds of responding. Increasing BMI was independently associated with reduced odds of responding to the alcohol question. Lastly, there was evidence (joint test $p=0.028$) that SBP was independently associated with the probability of missingness, with reduced odds of responding to the question for those with low or high SBP, relative to those with average SBP. Assuming that increasing levels of reported alcohol assumption is independently associated with increased SBP (see CCA results below), this finding is consistent with the probability that the alcohol variable is missing being elevated for those with either low or high alcohol consumption.

**Table 3.** Estimated adjusted odds ratios $(95\%$ CIs$)$ relating response to the alcohol question to age$,$ BMI and SBP in NHANES

ariable	Odds ratio (95% CI)	$p$-value
Age (decades above 50)	0.763 (0.723, 0.805)	$<$0.001
BMI (kg/m$^2$)	0.978 (0.961, 0.996)	0.019
SBP (per 10 mmHg above 125)	1.08 (1.01, 1.16)	0.020
SBP$^2$ (per 10 mmHg above 125)$^2$	0.979 (0.963, 0.996)	0.015

We fitted a linear regression model (using ordinary least squares and sandwich standard errors to allow for non-constant variance) for SBP with age (linear and quadratic effects), BMI, and $\log (\hbox {no. drinks} + 1)$ as covariates. The number of alcoholic drinks variable was entered using a (natural) log transformation so that the few participants with very large values did not have undue influence on parameter estimates and because preliminary analyses suggested a multiplicative effect of number of drinks fitted the data better. Table 4 shows the CCA estimates, which assuming missingness in the alcohol variable is independent of SBP, conditional on age, BMI, and reported average number of alcoholic drinks per day, are unbiased. There was strong evidence that, as expected, increasing age is associated with increased SBP, with some suggestion of a non-linear effect. Increasing BMI was associated with increasing SBP, and there was evidence that increasing reported alcohol consumption is associated with increasing SBP.

**Table 4.** Estimates of conditional mean model parameters relating SBP $($mmHg$)$ $($centered at $125$ mmHg$)$ to age$,$ BMI$,$ and reported average number of alcoholic drinks consumed per day in NHANES

	Variable				
Estimator	Constant	No. of drinks$^{\dagger }$	BMI (kg/m$^2$)	Age (decades above 50)	Age$^2$ (decades above 50)$^2$
CCA	$-$1.93 (0.80)	1.27 (0.58)	0.41 (0.080)	3.94 (0.26)	0.26 (0.14)
MI	$-$2.36 (0.81)	1.51 (0.65)	0.32 (0.070)	3.88 (0.21)	0.30 (0.12)
IPW	$-$2.07 (0.85)	1.50 (0.67)	0.36 (0.092)	3.95 (0.24)	0.21 (0.16)
$\hat {\beta }_{{\mathrm {ACC\hbox {-}}WM}}$	$-$2.21 (0.76)	1.40 (0.59)	0.39 (0.066)	3.90 (0.24)	0.32 (0.11)
$\hat {\beta }_{{\mathrm {ACC2\hbox {-}}WM}}$	$-$2.02 (0.75)	1.21 (0.58)	0.39 (0.065)	3.88 (0.24)	0.31 (0.11)
$\hat {\beta }_{{\mathrm {ACC\hbox {-}}NP}}$	$-$1.90 (0.94)	1.37 (0.67)	0.43 (0.107)	3.97 (0.31)	0.22 (0.21)
$\hat {\beta }_{{\mathrm {ACC\hbox {-}}NP2}}$	$-$2.03 (0.72)	1.20 (0.53)	0.39 (0.066)	3.87 (0.23)	0.32 (0.11)

Estimates from CCA, MI assuming MAR, IPW CCA assuming MAR, and ACC analysis using four different choices for $\phi (Y,Z,\beta)$ (see text for details).

$^{\dagger }$$\hbox {log}_{e}(\hbox {average no. drinks per day} +1)$.

Next we estimated the conditional mean model parameters assuming missingness in the alcohol variable was MAR, first using MI. The alcohol variable on its original scale was imputed 200 times using a negative binomial regression model with covariates age (linear and quadratic), BMI (linear and quadratic), and SBP (linear and quadratic). Standard errors were obtained using Rubin's rules, but using the sandwich estimator of variance when estimating within-imputation variances. Consistency of MI here relies on the MAR assumption holding and the imputation model being correctly specified. The resulting estimates were fairly similar to CCA, although the coefficient of BMI was somewhat lower, the coefficient of the alcohol variable was somewhat higher, and the estimated constant was lower than that from CCA. Standard errors were smaller than those from CCA for the effects of BMI and age.

Since consistency of MI relies on the imputation model being correctly specified, we also used complete case IPW, with weights calculated using the previously described logistic regression model. Sandwich standard errors were found by stacking the estimating equation used to estimate the parameters of this logistic regression with the IPW complete case estimating equations. The estimated linear age effect was similar to that from CCA, but the estimated quadratic effect was smaller. The estimated coefficient of BMI was slightly smaller than from CCA, and the estimated constant was closer to that from CCA than the MI estimate. The estimated coefficient of the alcohol variable was almost identical to the MI estimate. As is typical, the (sandwich) standard errors for IPW CCA were larger than those for CCA (except for the linear age coefficient).

Lastly, we used the proposed ACC estimator, using the logistic model shown in Table 3 for $P(R=1|Y,Z)$. We first used $\hat {\beta }_{{\mathrm {ACC\hbox {-}}WM}}$, with the parametric working model identical to that used to impute the alcohol variable in MI (i.e. negative binomial regression imputation). This gave estimates with smaller standard errors than CCA, and also lower than MI. The estimated constant and effect of alcohol were both in between the corresponding CCA and MI estimates. The estimated BMI effect was close to that from CCA, and the estimated age effects were similar to those from CCA. Using $\hat {\beta }_{{\mathrm {ACC2\hbox {-}}WM}}$ led to an estimated constant closer to that from CCA and an estimated effect of alcohol which was smaller than from CCA. Standard errors were very slightly smaller than from $\hat {\beta }_{{\rm ACC\hbox {-}WM}}$. The non-parametric estimator $\hat {\beta }_{{\mathrm {ACC\hbox {-}}NP}}$ gave estimates with much larger standard errors, whereas $\hat {\beta }_{{\rm ACC\hbox {-}NP2}}$ gave estimates with standard errors smaller than those from $\hat {\beta }_{{\rm ACC2\hbox {-}WM}}$.

Overall inferences from the methods were fairly similar. Nevertheless, standard errors were smallest from the proposed ACC estimator(s), and in particular were smaller than CCA for the effects of the fully observed covariates. There is the suggestion that the estimated effect of the alcohol variable was larger when assuming MAR compared with assuming missingness conditionally independent of outcome. Unlike in the simulations, there was no apparent substantial bias in the estimated constant from the methods which assumed MAR.

## 5. Discussion

In some settings, contextual knowledge will suggest that missingness in a covariate, such as income, is driven primarily by the value of the covariate itself, such that data are MNAR. In prospective studies, missingness in covariates measured at study entry may often plausibly be affected by the covariates themselves (and hence again MNAR), and given these, be independent of the (future) outcome. In these settings, an analysis based on the MAR assumption, such as most implementations of MI and IPW, will lead to asymptotically biased estimates and invalid inferences. For a linear conditional mean model, the analytical results of Appendix G of supplementary material available at *Biostatistics* online and simulation analyses suggest that the biases may be moderately large for the intercept parameter, but are sometimes modest for the parameters corresponding to covariate effects. However, there likely exist MNAR scenarios in which CCA is unbiased but MAR estimators of covariate effects are biased to a larger extent.

In contrast, if missingness is conditionally independent of outcome, which includes a particular class of MNAR mechanisms, CCA is unbiased but does not make use of all of the observed information. Our proposed augmented CCA estimator improves upon the efficiency of CCA, by relying on a parametric model for how missingness is associated with fully observed covariates and outcome. While one may argue whether it is appropriate to increase precision by relying on additional models, we note that this is also the case for other missing data methods. Furthermore, standard model selection techniques can be used since this model only involves fully observed variables. Given the assumption that missingness is independent of outcome given covariates, CCA and our proposed augmented CCA estimator are both consistent, provided the missingness model used in the latter is correctly specified.

Consistent with the findings of others (e.g. White and Carlin, 2010), in both our simulations and data analysis, while efficiency gain is possible for the coefficients of fully observed covariates, neither MI nor ACC gave improved efficiency for the covariate which was partially observed. This emphasizes the point that, in the absence of auxiliary variables or external information, the gain in efficiency (for both MI and ACC) is achieved through utilizing the observed information in incomplete cases. This implies that in studies where missingness only occurs in the exposure of interest, there is little efficiency to be gained for the exposure effect through using an estimation method which utilizes the incomplete cases.

For the proposed estimator, we have considered estimating the optimal augmentation function $\phi (Y,Z,\beta)$ either using a parametric working model or non-parametric kernel regression methods. For the latter, we found that direct non-parametric estimation of the optimal augmentation function lead to estimates with high variability, and in fact efficiency worse than CCA. In contrast, non-parametric estimation of the first two moments of the partially observed covariate, which is sufficient in the case of a linear conditional mean model, gave estimates with efficiency essentially identical to that obtained using the true optimal augmentation function. Further work is thus warranted regarding how to best non-parametrically estimate the optimal augmentation function directly for conditional mean models which are not linear.

The data analyst who adopts the assumption $R \perp \!\!\!\perp Y | X,Z$ should be aware of the fact that the observed data may sometimes carry evidence to refute it (see Appendix A of supplementary material available at *Biostatistics* online). This can be a concern in settings where the outcome is continuous,but the covariates are discrete with few levels (see, e.g. Vansteelandt, 2009 for an example where failure to study the testability of missing data assumptions lead to a severely biased analysis); however, we tend not to worry about it in more realistic settings where the power to refute the assumption will typically be very low. Further, the data analyst should consider whether their postulated model for $R|Y,Z$ is compatible with the restrictions imposed by the missing data assumption $R \perp \!\!\!\perp Y | X,Z$ together with the conditional mean model; careful checking of the missingness model is therefore recommended.

Throughout, we have restricted our development to the case of a single partially observed covariate or vector of covariates. However, we believe the approach may be extendable to more general patterns of missingness, including non-monotone patterns, and describe how this could be done for the case of two partially observed variables in Appendix H of supplementary material available at *Biostatistics* online. An advantage of such an approach would be that the missingness assumption $R \perp \!\!\!\perp Y |X,Z$, where $R$ is a vector of missingness indicators, is easier to interpret than MAR when missingness is non-monotone (Robins and Gill, 1997).

Lastly, we note that in the case of data MAR, so-called doubly robust estimators are available, which remain consistent so long as either the model for missingness or the imputation type model is correct (Tsiatis, 2006). The ACC estimator developed here does not possess such a doubly robust property, and it is indeed unclear whether such estimators exist under the assumptions considered here.

## 6. Software

A Stata program implementing $\hat {\beta }_{{\rm ACC2\hbox {-}WM}}$ for conditional linear mean models is available for free download by typing “net from http://missingdata.lshtm.ac.uk/stata” into Stata's command window and selecting “augcca”.

## Supplementary material

Supplementary material is available at http://biostatistics.oxfordjournals.org.

## Funding

This work was supported by UK Economic and Social Research Council [RES-189-25-0103 to J.W.B. and J.R.C.] and Medical Research Councils [G0900724 to J.W.B., J.R.C., and K.T., and MR/K02180X/1 to J.W.B.]. This work was also supported by the Interuniversity Attraction Poles Programme [P7/06 to SV]. Funding to pay the Open Access publication charges for this article was provided by the UK Medical Research Council.

## Supplementary Material

Supplementary Data
