# Supplementary material for: Improving upon the efficiency of complete case analysis when covariates are MNAR
Source: Biostatistics. 2014 Jun 6;15(4):719–30. doi: 10.1093/biostatistics/kxu023 (PMC4173105; doi:10.1093/biostatistics/kxu023)
Supplement: Supplementary Data [file supp_15_4_719__index.html]

Improving upon the efficiency of complete case analysis when covariates are MNAR — Improving upon the efficiency of complete case analysis when covariates are MNAR — Supplementary Data 

# Improving upon the efficiency of complete case analysis when covariates are MNAR

## Supplementary Data

Supplementary Data

**Files in this Supplementary Material:**

- Supplementary Data - Pdf file
