# Supplementary material for: Improving upon the efficiency of complete case analysis when covariates are MNAR
Source: Biostatistics. 2014 Jun 6;15(4):719–30. doi: 10.1093/biostatistics/kxu023 (PMC4173105; doi:10.1093/biostatistics/kxu023)
Supplement: Supplementary Data [file supp_kxu023_kxu023supp.pdf]

Biostatistics (2014), **0**, 0, *pp.* 1–26
doi:10.1093/biostatistics/BartlettSupplementaryMaterialsRevision2

Improving upon the efficiency of complete case analysis when covariates are MNAR Supplementary Materials

JONATHAN W. BARTLETT*

Centre for Statistical Methodology,

London School of Hygiene and Tropical Medicine,

Keppel Street, London, WC1E 7HT, UK

*jonathan.bartlett@lshtm.ac.uk

JAMES R. CARPENTER

Centre for Statistical Methodology,

London School of Hygiene and Tropical Medicine,

& MRC Clinical Trials Unit, Kingsway, London, WC2B 6NH, UK

KATE TILLING

School of Social and Community Medicine, University of Bristol,

Canynge Hall, 39 Whatley Road, Bristol, BS8 2PS, UK

STIJN VANSTEELANDT

Department of Applied Mathematics, Computer Science and Statistics,

Ghent University, Krijgslaan, 281 S9, B-9000 Ghent, Belgium

© The Author 2014. Published by Oxford University Press. All rights reserved. For permissions, please e-mail: journals.permissions@oup.com

APPENDIX

A. Testing the assumption that $R \perp\!\!\!\perp Y|X, Z$

In this appendix, we show that the complete case assumption that $R \perp\!\!\!\perp Y|X, Z$ may impose testable restrictions on the observed data, so that the model defined by the sole restriction $R \perp\!\!\!\perp Y|X, Z$ does not define a nonparametric identified model (Robins (1997)). This can be seen from the following example. Let Z be empty, X be dichotomous (0/1) and suppose that Y is discrete with three levels. We can then infer from the assumption that $Y \perp\!\!\!\perp R|X$ that

$$\begin{aligned} P(Y|R=0) &= P(Y|X=1, R=0)P(X=1|R=0) + P(Y|X=0, R=0)P(X=0|R=0) \\ &= P(Y|X=1)P(X=1|R=0) + P(Y|X=0)P(X=0|R=0). \end{aligned}$$

From the observed data $P(Y|R=0)$ and $P(Y|X=1, R=1) = P(Y|X=1)$ are identifiable for the three levels of Y, with $\pi = P(X=1|R=0)$ the only unknown. The preceding equation thus results in two restrictions from the first two levels taken by Y (the third equation following from the first and second) with only a single unknown, suggesting a testable restriction. The observed data may thus carry information which refute the (only) assumption that $R \perp\!\!\!\perp Y|X$, and so this assumption does not define a nonparametric identified model.

B. The plausibility of MAR

In some settings the assumption that $R \perp\!\!\!\perp Y|X, Z$ may be deemed plausible because of the timing of measurement of the variables. In prospective studies often the covariates X and Z are variables measured or recorded at entry to the study while Y is an outcome measured during follow-up. Since causes precede effects, it is not possible for Y to directly influence whether X is observed. Instead, missingness in X must be caused by some function of X, Z, and/or other factors (possibly unmeasured), U, which exist at the time of study entry. Assumptions about the causal relationships between variables can be represented using a directed acyclic graphs (DAG),

and as shown by Daniel *and others* (2012) these can be used to deduce whether the conditional independence assumptions for CCA validity or MAR hold.

First, consider the case where X and/or Z are the only causes of missingness in X (which also affect Y) (Figures 1a and 1b). In this case CCA is always valid, since $R \perp\!\!\!\perp Y|X, Z$. That the latter holds can be deduced by the rules for manipulating DAGs (Pearl (1995)), since after conditioning on X and Z, there exist no unblocked paths between R and Y. The MAR assumption will hold if missingness is caused by Z but not by X (as in Figure 1 (a)), but will not hold if missingness is caused by X (as in Figure 1 (b)).

Second, suppose there exist other (possibly unmeasured) variables U which cause missingness in X and which independently affect outcome Y, an example of which is shown in Figure 1 (c). In this case CCA will result in biased estimates, since Y and R will be dependent (conditional on X and Z) due to their common cause U. Even in situations, such as that depicted in Figure 1 (c), where X does not affect missingness in X, the MAR assumption is invalidated if U is unobserved, since conditioning on Y renders the common causes X and U dependent, inducing a dependence between R and X. Of course if U is observed and conditioned on, the MAR assumption would hold for the setting depicted in Figure 1 (c).

In conclusion, in some settings the MAR assumption may be implausible while an MNAR assumption in which missingness is conditionally independent of outcome, under which CCA is valid, may be reasonable. In other settings (e.g. shown in Figure 1 (c)) neither assumption may hold.

C. The necessity of additional assumptions to improve upon CCA efficiency

In this appendix we assume that a fully parametric model $f(Y|X, Z; \beta)$ is specified, where β is finite dimensional, and that $R \perp\!\!\!\perp Y|X, Z$. Suppose that no assumptions are made regarding the joint distribution $f(R, X, Z)$ and that the support of X is sufficiently large so that the complete

case assumption that $R \perp\!\!\!\perp Y|X, Z$ imposes no testable restrictions on the observed data. To characterise estimators of β in this semiparametric model we first identify the nuisance tangent space corresponding to η. The observed data likelihood for a single subject is

$$\{f(R=1,X,Z;\eta)f(Y|X,Z;\beta)\}^R \left\{\int f(R=0,X,Z;\eta)f(Y|X,Z;\beta)dX\right\}^{1-R}.$$

For each parametric submodel, the score vector with respect to the finite dimensional parameter η^{sm} indexing the model for $f(R,X,Z)$ is equal to

$$\begin{aligned}
&R\frac{\partial}{\partial\eta^{\text{sm}}}\log\{f(R=1,X,Z;\eta^{\text{sm}^*})\} + (1-R)\frac{\partial}{\partial\eta^{\text{sm}}}\log\left\{\int f(R=0,X,Z;\eta^{\text{sm}^*})f(Y|X,Z;\beta)dX\right\} \\
&= RS(R=1,X,Z) + (1-R)\frac{\int \frac{\partial}{\partial\eta^{\text{sm}}} f(R=0,X,Z;\eta^{\text{sm}^*})f(Y|X,Z;\beta)dX}{\int f(R=0,X,Z;\eta^{\text{sm}^*})f(Y|X,Z;\beta)dX} \\
&= RS(R=1,X,Z) + (1-R)E\{S(R=0,X,Z)|R=0,Y,Z\} \\
&= E\{S(R,X,Z)|O\}
\end{aligned}$$

where η^{sm^*} denotes the true value of η^{sm}, $S(R,X,Z) = \partial\log\{f(R,X,Z;\eta^{\text{sm}^*})\}/\partial\eta^{\text{sm}}$ at the particular submodel (and which therefore satisfies $E\{S(R,X,Z)\}=0$), and $O=(Y,Z,R,RX)^T$ denotes the observed data for a single subject. The nuisance tangent space is therefore equal to

$$\Lambda = [E\{a(R,X,Z)|O\} : E\{a(R,X,Z)\}=0].$$

Then the orthocomplement of the nuisance tangent space, denoted $\Lambda^\perp$, is the set of functions $b(O)$ that satisfy

$$E\left[b^T(O)E\{a(R,X,Z)|O\}\right] = 0$$

for all mean zero functions $a(R,X,Z)$. It follows that

$$\begin{aligned}
0 &= E\left[E\left\{b^T(O)a(R,X,Z)|O\right\}\right] \\
&= E\left[b^T(O)a(R,X,Z)\right] \\
&= E\left[E\{b(O)|R,X,Z\}^T a(R,X,Z)\right]
\end{aligned}$$

so that $E\{b(O)|R,X,Z\}$ must be contained in the orthocomplement of the set of mean zero functions $a(R,X,Z)$. Upon writing $b(O)=Rb_1(Y,X,Z)+(1-R)b_0(Y,Z)$, we thus find that

$$\begin{aligned}\Lambda^{\perp} &= [Rb_1(Y,X,Z)+(1-R)b_0(Y,Z) : E\{Rb_1(Y,X,Z)+(1-R)b_0(Y,Z)|R,X,Z\}=0] \\ &= [Rb_1(Y,X,Z)+(1-R)b_0(Y,Z) : RE\{b_1(Y,X,Z)|R=1,X,Z\} \\ &\quad +(1-R)E\{b_0(Y,Z)|R=0,X,Z\}=0] \\ &= [Rb_1(Y,X,Z)+(1-R)b_0(Y,Z) : RE\{b_1(Y,X,Z)|X,Z\}+(1-R)E\{b_0(Y,Z)|X,Z\}=0] \\ &= [Rb_1(Y,X,Z)+(1-R)b_0(Y,Z) : E\{b_1(Y,X,Z)|X,Z\}=0, E\{b_0(Y,Z)|X,Z\}=0]\end{aligned}$$

Under a parametric model for $f(Y|X,Z)$, it is easy to find functions $b_1(Y,X,Z)$ that satisfy $E\{b_1(Y,X,Z)|X,Z\}=0$, namely all functions $b(Y,X,Z)-E\{b(Y,X,Z)|X,Z\}$. If $Y \perp\!\!\!\perp X|Z$, we can similarly define functions $b_0(Y,Z)=b(Y,Z)-E\{b(Y,Z)|Z\}$ which satisfy $E\{b_0(Y,Z)|X,Z\}=0$, since $Y \perp\!\!\!\perp X|Z$ implies $E(b(Y,Z)|Z)=E(b(Y,Z)|X,Z)$. Otherwise, unless assumptions are made about the distribution of $X|Z$, this condition can only be satisfied if $b_0(Y,Z)=0$, such that $b(O)$ is a function only of data from complete cases. Since the influence function of any estimator of β must belong to $\Lambda^{\perp}$, we conclude that such estimators only make use of information from the complete cases. Therefore to be able to extract information from the incomplete cases, additional modelling assumptions must be made.

D. Influence function and efficiency

D.1 *Influence function*

In this appendix we first derive the influence function of the estimator $\hat{\beta}_{\text{ACC}}$ which solves

$$\sum_{i=1}^{n} m(O_i, \hat{\alpha}, \hat{\beta}_{\text{ACC}}) = 0$$

Under suitable regularity conditions, we can expand the estimating equation about β^* so that

$$\begin{aligned} n^{1/2}(\hat{\beta}_{\text{ACC}} - \beta^*) &= -\left\{ n^{-1} \sum_{i=1}^{n} \frac{\partial m(O_i, \hat{\alpha}, \tilde{\beta})}{\partial \beta^T} \right\}^{-1} n^{-1/2} \sum_{i=1}^{n} m(O_i, \hat{\alpha}, \beta^*) \\ &= -\left\{ E\left[\frac{\partial m(O, \alpha^*, \beta^*)}{\partial \beta^T} \right] \right\}^{-1} n^{-1/2} \sum_{i=1}^{n} m(O_i, \hat{\alpha}, \beta^*) + o_p(1) \end{aligned}$$

where $\tilde{\beta}$ is an intermediate value between $\hat{\beta}_{\text{ACC}}$ and β^* and $o_p(1)$ is a term that converges in probability to zero as n tends to infinity. Expanding next around α^*, and letting $G_\beta = E\left[\partial m(O, \alpha^*, \beta^*)/\partial \beta^T\right]$, we obtain

$$\begin{aligned} n^{1/2}(\hat{\beta}_{\text{ACC}} - \beta^*) &= -G_\beta^{-1} \left[n^{-1/2} \sum_{i=1}^{n} m(O_i, \alpha^*, \beta^*) + \left\{ n^{-1} \sum_{i=1}^{n} \frac{\partial m(O_i, \tilde{\alpha}, \beta^*)}{\partial \alpha^T} \right\} n^{1/2}(\hat{\alpha} - \alpha^*) \right] \\ &\quad + o_p(1) \\ &= -G_\beta^{-1} \left[n^{-1/2} \sum_{i=1}^{n} m(O_i, \alpha^*, \beta^*) + E\left\{ \frac{\partial m(O_i, \alpha^*, \beta^*)}{\partial \alpha^T} \right\} n^{1/2}(\hat{\alpha} - \alpha^*) \right] + o_p(1) \end{aligned}$$

where $\tilde{\alpha}$ denotes an intermediate value between $\hat{\alpha}$ and α^*. Let $\varphi_\alpha(Y, Z, R)$ denote the influence function of $\hat{\alpha}$, i.e. the mean (at α^*) zero function that satisfies

$$n^{1/2}(\hat{\alpha} - \alpha^*) = n^{-1/2} \sum_{i=1}^{n} \varphi_\alpha(Y_i, Z_i, R_i) + o_p(1)$$

Then letting $G_\alpha = E\left[\partial m(O, \alpha^*, \beta^*)/\partial \alpha^T\right]$, we have that

$$n^{1/2}(\hat{\beta}_{\text{ACC}} - \beta^*) = -G_\beta^{-1} \left\{ n^{-1/2} \sum_{i=1}^{n} m(O_i, \alpha^*, \beta^*) + G_\alpha \varphi_\alpha(Y_i, Z_i, R_i) \right\} + o_p(1)$$

and thus that the influence function of $\hat{\beta}_{\text{ACC}}$ is equal to

$$-G_\beta^{-1}\{m(O, \alpha^*, \beta^*) + G_\alpha \varphi_\alpha(Y, Z, R)\}$$

The matrix G_β is equal to

$$\begin{aligned} G_\beta &= E\left[\frac{\partial}{\partial \beta^T} m(O, \alpha^*, \beta^*) \right] \\ &= E\left[\frac{\partial}{\partial \beta^T} \left\{ Rd(X, Z)\epsilon(\beta^*) + \{R - \pi(Y, Z; \alpha^*)\}\phi(Y, Z, \beta^*) \right\} \right] \\ &= E\left[\frac{\partial}{\partial \beta^T} Rd(X, Z)\epsilon(\beta^*) \right] \qquad \text{since } E\left[\{R - \pi(Y, Z; \alpha^*)\} \frac{\partial}{\partial \beta^T} \phi(Y, Z, \beta^*) | Y, Z \right] = 0 \end{aligned}$$

The matrix G_α is equal to

$$\begin{aligned} G_\alpha &= E\left[\frac{\partial}{\partial\alpha^T} m(O,\alpha^*,\beta^*)\right] \\ &= E\left[\frac{\partial}{\partial\alpha^T}\left\{Rd(X,Z)\epsilon(\beta^*) + \{R-\pi(Y,Z;\alpha^*)\}\phi(Y,Z,\beta^*)\right\}\right] \\ &= -E\left[\frac{\partial}{\partial\alpha^T}\pi(Y,Z;\alpha^*)\phi(Y,Z,\beta^*)\right] \\ &= -E\left[\pi(Y,Z;\alpha^*)(1-\pi(Y,Z;\alpha^*))\phi(Y,Z,\beta^*)h_\alpha^T(Y,Z)\right] \end{aligned}$$

and lastly the influence function $\varphi_\alpha(Y,Z,R)$ is given by

$$\begin{aligned} \varphi_\alpha(Y,Z,R) &= -\left[E\left\{\frac{\partial}{\partial\alpha^T}(R-\pi(Y,Z;\alpha^*))h_\alpha(Y,Z)\right\}\right]^{-1}\{R-\pi(Y,Z;\alpha^*)\}h_\alpha(Y,Z) \\ &= \left[E\left\{\pi(Y,Z;\alpha^*)(1-\pi(Y,Z;\alpha^*))h_\alpha(Y,Z)h_\alpha^T(Y,Z)\right\}\right]^{-1} \\ &\quad \times\{R-\pi(Y,Z;\alpha^*)\}h_\alpha(Y,Z) \end{aligned}$$

The influence function of $\hat{\beta}_{\text{ACC}}$ can thus be written as

$$-G_\beta^{-1}\left[Rd(X,Z)\epsilon(\beta^*) + \{R-\pi(Y,Z;\alpha^*)\}\tilde{\phi}(Y,Z,\alpha^*,\beta^*)\right]$$

where

$$\begin{aligned} \tilde{\phi}(y,z,\alpha,\beta) = \phi(y,z,\beta) &- E\left[\pi(Y,Z;\alpha)\{1-\pi(Y,Z;\alpha)\}\phi(Y,Z,\beta)h_\alpha^T(Y,Z)\right] \\ &\times\left\{E\left[\pi(Y,Z;\alpha)\{1-\pi(Y,Z;\alpha)\}h_\alpha(Y,Z)h_\alpha^T(Y,Z)\right]\right\}^{-1}h_\alpha(y,z) \end{aligned}$$

D.2 *Efficiency*

We now consider the efficiency of $\hat{\beta}_{\text{INF}}$ and $\hat{\beta}_{\text{ACC}}$. The efficiency of these estimators is affected by the choices of the functions $d(X,Z)$ and $\phi(Y,Z,\beta)$. For simplicity, rather than attempt to find the optimal choices for both of these functions, we consider the optimal choice of $\phi(Y,Z,\beta)$ for a fixed choice of $d(X,Z)$ (e.g. at the choice we would use with full data).

D.2.1 *α known* First, consider the optimal choice of $\phi(Y,Z,\beta)$ for the estimator $\hat{\beta}_{\text{INF}}$ which uses the true value α^*. The asymptotic variance of the estimator is equal to the variance of

the influence function, which equals $G_\beta^{-1}\text{Var}\left\{m(O,\alpha^*,\beta^*)\right\}{G_\beta^{-1}}^T$. Since G_β is unaffected by the choice of $\phi(Y,Z,\beta)$, the choice of $\phi(Y,Z,\beta)$ only affects the variance of $\hat{\beta}_{\text{INF}}$ through the variance of the estimating function $m(O,\alpha^*,\beta^*)$. Let $\pi(Y,Z)=\pi(Y,Z;\alpha^*)$, $\phi(Y,Z)=\phi(Y,Z,\beta^*)$, and $\epsilon=\epsilon(\beta^*)$. Then we define

$$\Lambda_\alpha^\perp = [\{R-\pi(Y,Z)\}\phi(Y,Z) : \phi(Y,Z) \text{ arbitrary}]$$

Then for a given function $d(X,Z)$, we seek the element in $\Lambda_\alpha^\perp$ which results in $m(O,\alpha^*,\beta^*)$ having smallest (in the positive definite sense) variance. We let $\{R-\pi(Y,Z)\}\phi_{\text{INF}}^{\text{opt}}(Y,Z)$ denote this element. Since $\Lambda_\alpha^\perp$ is a closed linear subspace, the projection of $Rd(X,Z)\epsilon$ onto $\Lambda_\alpha^\perp$ exists. It is easy to show that $Rd(X,Z)\epsilon$ minus its projection onto $\Lambda_\alpha^\perp$ results in $m(O,\alpha^*,\beta^*)$ having minimum variance. Thus

$$\{R-\pi(Y,Z)\}\phi_{\text{INF}}^{\text{opt}}(Y,Z) = -\prod\left\{Rd(X,Z)\epsilon|\Lambda_\alpha^\perp\right\}$$

where $\prod$ denotes the orthogonal projection operator with respect to the covariance inner product. The residual of the projection must be orthogonal to all elements in $\Lambda_\alpha^\perp$, and so

$$0 = E\left[(R-\pi(Y,Z))\phi^T(Y,Z)\left\{Rd(X,Z)\epsilon + (R-\pi(Y,Z))\phi_{\text{INF}}^{\text{opt}}(Y,Z)\right\}\right]$$

for all functions $\phi(Y,Z)$. This holds if and only if

$$\begin{aligned}0 &= E\left[(R-\pi(Y,Z))\left\{Rd(X,Z)\epsilon + (R-\pi(Y,Z))\phi_{\text{INF}}^{\text{opt}}(Y,Z)\right\}|Y,Z\right]\\ &= E\left[(1-\pi(Y,Z))Rd(X,Z)\epsilon|Y,Z\right] + E\left[(R-\pi(Y,Z))^2\phi_{\text{INF}}^{\text{opt}}(Y,Z)|Y,Z\right]\\ &= (1-\pi(Y,Z))E\left[Rd(X,Z)\epsilon|Y,Z\right] + \pi(Y,Z)(1-\pi(Y,Z))\phi_{\text{INF}}^{\text{opt}}(Y,Z)\end{aligned}$$

and so

$$\begin{aligned}\phi_{\text{INF}}^{\text{opt}}(Y,Z) &= -\frac{E\left[Rd(X,Z)\epsilon|Y,Z\right]}{\pi(Y,Z)}\\ &= -E\left[d(X,Z)\epsilon|R=1,Y,Z\right] \qquad \text{(D.1)}\end{aligned}$$

The estimator which uses $\phi^{\mathrm{opt}}(Y,Z,\beta) = -E\left[d(X,Z)\epsilon(\beta)|R=1,Y,Z\right]$ then attains the minimum variance.

D.2.2 *α estimated* Now consider the optimal choice of $\phi(Y,Z,\beta)$ for $\hat{\beta}_{\mathrm{ACC}}$, which uses $\hat{\alpha}$. For a given choice $\phi(Y,Z,\beta)$, the influence function of $\hat{\beta}_{\mathrm{ACC}}$ is proportional to

$$Rd(X,Z)\epsilon + \{R-\pi(Y,Z)\}\tilde{\phi}(Y,Z)$$

where $\tilde{\phi}(Y,Z) = \tilde{\phi}(Y,Z,\alpha^*,\beta^*)$. Let

$$\Sigma_{\phi\alpha} = E\left[\pi(Y,Z)\{1-\pi(Y,Z)\}\phi(Y,Z)h_\alpha^T(Y,Z)\right]$$

and

$$\Sigma_\alpha = E\left[\pi(Y,Z)\{1-\pi(Y,Z)\}h_\alpha(Y,Z)h_\alpha^T(Y,Z)\right]$$

so that we can more succinctly write

$$\tilde{\phi}(Y,Z) = \phi(Y,Z) - \Sigma_{\phi\alpha}\Sigma_\alpha^{-1}h_\alpha(Y,Z)$$

Let

$$\tilde{\Lambda}_\alpha^\perp = \left[\{R-\pi(Y,Z)\}\,\tilde{\phi}(Y,Z) : \phi(Y,Z) \text{ arbitrary}\right]$$

Then we seek the function $\phi_{ACC}^{\mathrm{opt}}(Y,Z)$, which induces the element $\{R-\pi(Y,Z)\}\,\tilde{\phi}_{ACC}^{\mathrm{opt}}(Y,Z) \in \tilde{\Lambda}_\alpha^\perp$, such that the variance of the influence function of $\hat{\beta}_{\mathrm{ACC}}$ is minimized. Following the corresponding proof for $\hat{\beta}_{\mathrm{INF}}$, this is achieved by choosing $\phi_{ACC}^{\mathrm{opt}}(Y,Z)$ such that

$$\{R-\pi(Y,Z)\}\,\tilde{\phi}_{ACC}^{\mathrm{opt}}(Y,Z) = -\prod\left\{Rd(X,Z)\epsilon|\tilde{\Lambda}_\alpha^\perp\right\}$$

Since $\tilde{\Lambda}_\alpha^\perp \subset \Lambda_\alpha^\perp$

$$\prod\left\{Rd(X,Z)\epsilon|\tilde{\Lambda}_\alpha^\perp\right\} = \prod\left[\prod\left\{Rd(X,Z)\epsilon|\Lambda_\alpha^\perp\right\}|\tilde{\Lambda}_\alpha^\perp\right]$$

We previously showed that

$$\prod \left\{ Rd(X,Z)\epsilon | \Lambda_\alpha^\perp \right\} = -\left\{ R - \pi(Y,Z) \right\} \phi_{\mathrm{INF}}^{\mathrm{opt}}(Y,Z)$$

Therefore

$$\prod \left\{ Rd(X,Z)\epsilon | \tilde{\Lambda}_\alpha^\perp \right\} = \prod \left[-\left\{ R - \pi(Y,Z) \right\} \phi_{\mathrm{INF}}^{\mathrm{opt}}(Y,Z) | \tilde{\Lambda}_\alpha^\perp \right]$$

The residual of the projection must be orthogonal to all elements in $\tilde{\Lambda}_\alpha^\perp$, and so is the unique element satisfying

$$\begin{aligned}
0 &= E\left[(R-\pi(Y,Z))\tilde{\phi}^T(Y,Z) \left\{ -\left\{ R - \pi(Y,Z) \right\} \phi_{\mathrm{INF}}^{\mathrm{opt}}(Y,Z) + \left\{ R - \pi(Y,Z) \right\} \tilde{\phi}_{ACC}^{\mathrm{opt}}(Y,Z) \right\} \right] \\
&= E\left[(R-\pi(Y,Z))^2 \tilde{\phi}^T(Y,Z) \left\{ \tilde{\phi}_{ACC}^{\mathrm{opt}}(Y,Z) - \phi_{\mathrm{INF}}^{\mathrm{opt}}(Y,Z) \right\} \right] \\
&= E\left[\pi(Y,Z)(1-\pi(Y,Z))\tilde{\phi}^T(Y,Z) \left\{ \tilde{\phi}_{ACC}^{\mathrm{opt}}(Y,Z) - \phi_{\mathrm{INF}}^{\mathrm{opt}}(Y,Z) \right\} \right] \qquad \text{(D.2)}
\end{aligned}$$

where $\tilde{\phi}(Y,Z)$ is defined via an arbitrary function $\phi(Y,Z)$.

We will now show that this is satisfied by choosing $\phi_{ACC}^{\mathrm{opt}}(Y,Z) = \phi_{\mathrm{INF}}^{\mathrm{opt}}(Y,Z)$. For this choice we have

$$\tilde{\phi}_{ACC}^{\mathrm{opt}}(Y,Z) = \phi_{\mathrm{INF}}^{\mathrm{opt}}(Y,Z) - \Sigma_{\phi_{\mathrm{INF}}^{\mathrm{opt}}} \Sigma_\alpha^{-1} h_\alpha(Y,Z)$$

Then the right hand side of equation (D.2) is equal to

$$E\left[\pi(Y,Z)(1-\pi(Y,Z))\tilde{\phi}^T(Y,Z) \left\{ -\Sigma_{\phi_{\mathrm{INF}}^{\mathrm{opt}}} \Sigma_\alpha^{-1} h_\alpha(Y,Z) \right\} \right] \qquad \text{(D.3)}$$

To show this equals zero, consider instead

$$\begin{aligned}
& E\left[\pi(Y,Z)(1-\pi(Y,Z))\tilde{\phi}(Y,Z) \left\{ h_\alpha^T(Y,Z)\Sigma_\alpha^{-1}\Sigma_{\phi_{\mathrm{INF}}^{\mathrm{opt}}}^T \right\} \right] \\
&= E\left[\pi(Y,Z)(1-\pi(Y,Z)) \left\{ \phi(Y,Z) - \Sigma_{\phi\alpha}\Sigma_\alpha^{-1} h_\alpha(Y,Z) \right\} \left\{ h_\alpha^T(Y,Z)\Sigma_\alpha^{-1}\Sigma_{\phi_{\mathrm{INF}}^{\mathrm{opt}}}^T \right\} \right] \\
&= E\left[\pi(Y,Z)(1-\pi(Y,Z))\phi(Y,Z)h_\alpha^T(Y,Z) \right] \Sigma_\alpha^{-1}\Sigma_{\phi_{\mathrm{INF}}^{\mathrm{opt}}}^T \\
&\quad -\Sigma_{\phi\alpha}\Sigma_\alpha^{-1} E\left[\pi(Y,Z)(1-\pi(Y,Z))h_\alpha(Y,Z)h_\alpha^T(Y,Z) \right] \Sigma_\alpha^{-1}\Sigma_{\phi_{\mathrm{INF}}^{\mathrm{opt}}}^T \\
&= \Sigma_{\phi\alpha}\Sigma_\alpha^{-1}\Sigma_{\phi_{\mathrm{INF}}^{\mathrm{opt}}}^T - \Sigma_{\phi\alpha}\Sigma_\alpha^{-1}\Sigma_{\phi_{\mathrm{INF}}^{\mathrm{opt}}}^T \\
&= 0
\end{aligned}$$

Since in particular the diagonals of this matrix are all zero, this implies that the expression in equation (D.3) is equal to zero. We thus conclude that $\phi^{\text{opt}}_{ACC}(Y,Z) = \phi^{\text{opt}}_{\text{INF}}(Y,Z)$, so that the optimal choice of $\phi(Y,Z,\beta)$ when α must be estimated is the same as the optimal choice when α is known.

D.3 *Guaranteeing an efficiency improvement over CCA*

If the working model used to estimate $\phi^{\text{opt}}(Y,Z,\beta)$ is mis-specified, $\hat{\beta}_{\text{ACC}}$ could be less efficient than $\hat{\beta}_{CCA}$. Here we derive an estimator $\hat{\beta}_{\text{ACC2}}$ which is guaranteed to be at least as efficient as $\hat{\beta}_{CCA}$, for a given choice of $\phi(Y,Z,\beta)$ (or working model used to estimate $\phi^{\text{opt}}(Y,Z,\beta)$). To guarantee an efficiency improvement over CCA, consider solving β using an estimator with influence function

$$-G_{\beta}^{-1}\left[Rd(X,Z)\epsilon(\beta^*) + \lambda\left\{R - \pi(Y,Z;\alpha^*)\right\}\tilde{\phi}(Y,Z,\alpha^*,\beta^*)\right] \tag{D.4}$$

where λ is a constant square matrix with the same number of rows as β. Then the variance of this influence function is minimized by choosing $\lambda = \lambda(\alpha^*,\beta^*)$, where

$$\begin{aligned}\lambda(\alpha,\beta) = -E&\left[Rd(X,Z)\epsilon(\beta)\left\{R - \pi(Y,Z;\alpha)\right\}\tilde{\phi}(Y,Z,\alpha,\beta)\right]\\ &\times\left\{E\left[\left\{R - \pi(Y,Z,\alpha)\right\}^2\tilde{\phi}(Y,Z,\alpha,\beta)\tilde{\phi}^T(Y,Z,\alpha,\beta)\right]\right\}^{-1}\end{aligned} \tag{D.5}$$

Since $\lambda = 0$ and $\lambda = I$ (the identity matrix) correspond to the influence functions of $\hat{\beta}_{CCA}$ and $\hat{\beta}_{\text{ACC}}$ respectively, an estimator with influence function given by (D.4) with $\lambda = \lambda(\alpha^*,\beta^*)$ will have a smaller asymptotic variance (in the positive definite sense) than both $\hat{\beta}_{CCA}$ and $\hat{\beta}_{\text{ACC}}$. Following the previous derivation of the influence function of $\hat{\beta}_{\text{ACC}}$, it is easy to show that the estimator of β with estimating function

$$Rd(X,Z)\epsilon(\beta) + \lambda(\alpha^*,\beta^*)\{R - \pi(Y,Z;\hat{\alpha})\}\phi(Y,Z,\beta) \tag{D.6}$$

has the desired influence function. Of course this estimator is not feasible, since the value $\lambda = \lambda(\alpha^*, \beta^*)$ is unknown. However, $\lambda(\alpha^*, \beta^*)$ can be consistently estimated by $\hat{\lambda}(\hat{\alpha}, \hat{\beta}_{CCA})$, where

$$\hat{\lambda}(\alpha,\beta) = -\left[\sum_{i=1}^{n} R_i d(X_i, Z_i)\epsilon(\beta)\left\{R_i - \pi(Y_i, Z_i; \alpha)\right\}\hat{\tilde{\phi}}(Y_i, Z_i, \alpha, \beta)\right] \times \left[\sum_{i=1}^{n} \{R_i - \pi(Y_i, Z_i, \alpha)\}^2 \hat{\tilde{\phi}}(Y_i, Z_i, \alpha, \beta)\hat{\tilde{\phi}}^T(Y_i, Z_i, \alpha, \beta)\right]^{-1}$$

and

$$\hat{\tilde{\phi}}(Y, Z, \alpha, \beta) = \phi(Y, Z, \beta) - \left[n^{-1}\sum_{i=1}^{n} \pi(Y_i, Z_i; \alpha)\{1 - \pi(Y_i, Z_i; \alpha)\}\phi(Y_i, Z_i, \beta)h_\alpha^T(Y_i, Z_i)\right] \times \left[n^{-1}\sum_{i=1}^{n} \pi(Y_i, Z_i; \alpha)\{1 - \pi(Y_i, Z_i; \alpha)\}h_\alpha(Y_i, Z_i)h_\alpha^T(Y_i, Z_i)\right]^{-1} h_\alpha(Y, Z)$$

Finally, β can be estimated as the value $\hat{\beta}_{\text{ACC2}}$ which satisfies

$$\sum_{i=1}^{n} R_i d(X_i, Z_i)\epsilon_i(\hat{\beta}_{\text{ACC2}}) + \hat{\lambda}(\hat{\alpha}, \hat{\beta}_{CCA})\{R_i - \pi(Y_i, Z_i; \hat{\alpha})\}\phi(Y_i, Z_i, \hat{\alpha}, \hat{\beta}_{\text{ACC2}}) = 0 \qquad \text{(D.7)}$$

Since the choice of λ only affects the variance of the estimator of β, and not its consistency, by Theorem 6.2 of Newey and McFadden (1994) $\hat{\beta}_{\text{ACC2}}$ has the same variance as the estimator with estimating function given in equation (D.6), i.e. when the value $\lambda(\alpha^*, \beta^*)$ is known. This also means that uncertainty in the estimate $\hat{\lambda}(\hat{\alpha}, \hat{\beta}_{CCA})$ can be ignored when calculating variance estimates for $\hat{\beta}_{\text{ACC2}}$.

E. Asymptotic distribution of $\hat{\beta}_{\text{ACC}-\text{NP}}$

For notational convenience, let $F_i = (Y_i, Z_i)$ denote the fully observed variables, $\phi^{\text{opt}}(F_i) = E\left\{d(X_i, Z_i)\epsilon_i(\beta)|R_i = 1, F_i\right\}$ and

$$\hat{\phi}^{\text{opt}}(F_i) = \frac{\sum_{j=1}^{n} R_j d(X_j, Z_j)\epsilon_j(\hat{\beta}_{CCA})K_h(F_i - F_j)}{\sum_{j=1}^{n} R_j K_h(F_i - F_j)},$$

where $\hat{\beta}_{CCA}$ denotes the complete case estimator of β. We then have that (where here $\hat{\beta}$ denotes $\hat{\beta}_{\text{ACC-NP}}$)

$$
\begin{aligned}
0 &= \frac{1}{\sqrt{n}}\sum_{i=1}^{n} R_i d(X_i,Z_i)\epsilon_i(\hat{\beta}) + \frac{1}{\sqrt{n}}\sum_{i=1}^{n}\{R_i - \pi(F_i;\hat{\alpha})\}\,\hat{\phi}^{\text{opt}}(F_i) \\
&= \frac{1}{\sqrt{n}}\sum_{i=1}^{n} R_i d(X_i,Z_i)\epsilon_i(\beta^*) + \left\{\frac{1}{n}\sum_{i=1}^{n} R_i d(X_i,Z_i)\frac{\partial\epsilon_i(\tilde{\beta})}{\partial\beta}\right\}\sqrt{n}(\hat{\beta}-\beta^*) \\
&+ \frac{1}{\sqrt{n}}\sum_{i=1}^{n}\{R_i - \pi(F_i;\alpha^*)\}\,\phi^{\text{opt}}(F_i) + \frac{1}{\sqrt{n}}\sum_{i=1}^{n}\{\pi(F_i;\alpha^*) - \pi(F_i;\hat{\alpha})\}\,\phi^{\text{opt}}(F_i) \\
&+ \frac{1}{\sqrt{n}}\sum_{i=1}^{n}\{R_i - \pi(F_i;\hat{\alpha})\}\left\{\hat{\phi}^{\text{opt}}(F_i) - \phi^{\text{opt}}(F_i)\right\} \\
&= \frac{1}{\sqrt{n}}\sum_{i=1}^{n} R_i d(X_i,Z_i)\epsilon_i(\beta^*) + \left\{\frac{1}{n}\sum_{i=1}^{n} R_i d(X_i,Z_i)\frac{\partial\epsilon_i(\tilde{\beta})}{\partial\beta}\right\}\sqrt{n}(\hat{\beta}-\beta^*) \\
&+ \frac{1}{\sqrt{n}}\sum_{i=1}^{n}\{R_i - \pi(F_i;\alpha^*)\}\,\phi^{\text{opt}}(F_i) - \left\{\frac{1}{n}\sum_{i=1}^{n}\frac{\partial\pi}{\partial\alpha}(F_i;\tilde{\alpha})\phi^{\text{opt}}(F_i)\right\}\sqrt{n}(\hat{\alpha}-\alpha^*) \\
&+ \frac{1}{\sqrt{n}}\sum_{i=1}^{n}\{R_i - \pi(F_i;\alpha^*)\}\left\{\hat{\phi}^{\text{opt}}(F_i) - \phi^{\text{opt}}(F_i)\right\} \\
&- \frac{1}{n}\sum_{i=1}^{n}\left[\frac{\partial\pi}{\partial\alpha}(F_i;\tilde{\alpha})\left\{\hat{\phi}^{\text{opt}}(F_i) - \phi^{\text{opt}}(F_i)\right\}\right]\sqrt{n}(\hat{\alpha}-\alpha^*).
\end{aligned}
$$

where $\tilde{\alpha}$ $(\tilde{\beta})$ is an intermediate value between α^* (β^*) and $\hat{\alpha}$ $(\hat{\beta})$. Suppose, as we assume throughout, that $\pi(F_i;\alpha) = \text{expit}\{w(F_i)\alpha\}$ for some covariate vector $w(F_i)$. Then

$$
\sup_{\tilde{\alpha}} \left|\left|\frac{1}{n}\sum_{i=1}^{n}\frac{\partial\pi}{\partial\alpha}(F_i;\tilde{\alpha})\left\{\hat{\phi}^{\text{opt}}(F_i) - \phi^{\text{opt}}(F_i)\right\}\right|\right| \leqslant \frac{1}{4n}\sum_{i=1}^{n}\left|\left|w(F_i)\left\{\hat{\phi}^{\text{opt}}(F_i) - \phi^{\text{opt}}(F_i)\right\}\right|\right|.
$$

We will now show that the latter expression is $o_p(1)$ and that, moreover,

$$
\frac{1}{\sqrt{n}}\sum_{i=1}^{n}\{R_i - \pi(F_i;\alpha^*)\}\left\{\hat{\phi}^{\text{opt}}(F_i) - \phi^{\text{opt}}(F_i)\right\} = o_p(1).
$$

It follows that $\hat{\beta}_{\text{ACC-TRUE}}$ and $\hat{\beta}_{\text{ACC-NP}}$ have the same asymptotic variance, and thus that there is no need to account for kernel estimation of $\phi^{\text{opt}}(F_i)$.

Let r denote the number of continuous components in F and define

$$\hat{B}_{ni} = \frac{1}{nh^r}\sum_{j=1}^{n} R_j d(X_j, Z_j)\epsilon_j(\hat{\beta}_{CCA})K_h(F_i - F_j)$$
$$= B_{ni} + \frac{1}{nh^r}\sum_{j=1}^{n} R_j d(X_j, Z_j)\frac{\partial \epsilon_j(\tilde{\beta})}{\partial \beta}K_h(F_i - F_j)(\hat{\beta}_{CCA} - \beta^*)$$

with $B_{ni} \equiv B_n(F_i)$,

$$B_n(F) = \frac{1}{nh^r}\sum_{j=1}^{n} R_j d(X_j, Z_j)\epsilon_j(\beta^*)K_h(F - F_j)$$

and $B(F) = E\{Rd(X,Z)\epsilon(\beta^*)|F\} f_F(F)$. Let $||.||$ be the Sobolev-norm of order 2. Then it follows from Lemma 8.10 in Newey and McFadden (1994) (under regularity assumptions 8.1 to 8.3 in Newey and McFadden (1994)) that

$$||B_n(O) - B(O)|| = O_p\left\{(\log n)^{1/2}(nh^{r+4})^{-1/2} + h^m\right\},$$

where m denotes the order of the kernel function. Let $\epsilon_i(\beta) = Y_i - g\{W(F_i)\beta\}$ for a covariate vector $W(F_i)$ and assume that

$$\sup_{\beta} ||\sum_{j=1}^{n} R_j d(X_j, Z_j)\frac{\partial \epsilon_j(\beta)}{\partial \beta}K_h(F_i - F_j)|| \leqslant M\sum_{j=1}^{n} ||R_j d(X_j, Z_j)W(F_i)K_h(F_i - F_j)||,$$

for a finite constant M, which is trivially satisfied for linear and logistic regression. By a similar reasoning as for $B_n(F)$, the fact that $||\hat{\beta}_{CCA} - \beta^*|| = O_p(n^{-1/2})$ and the triangle inequality, we then obtain that also

$$||\hat{B}_n(F) - B(F)|| = O_p\left\{(\log n)^{1/2}(nh^{r+4})^{-1/2} + h^m\right\}.$$

Likewise, let

$$A_n(F) = \frac{1}{nh^r}\sum_{j=1}^{n} R_j K_h(F - F_j)$$

and $A(F) = E(R|F) f_F(F)$. It is then immediate from Newey and McFadden (1994) that

$$||A_n(F) - A(F)|| = O_p\left\{(\log n)^{1/2}(nh^{r+4})^{-1/2} + h^m\right\}. \tag{E.1}$$

Now,

$$\frac{1}{\sqrt{n}}\sum_{i=1}^{n}\{R_i-\pi(F_i;\alpha^*)\}\left\{\hat{\phi}^{\text{opt}}(F_i)-\phi^{\text{opt}}(F_i)\right\}$$
$$=\frac{1}{\sqrt{n}}\sum_{i=1}^{n}\frac{\{R_i-\pi(F_i;\alpha^*)\}}{A(F_i)}\left\{\hat{B}_n(F_i)-\phi^{\text{opt}}(F_i)A_n(F_i)-B(F_i)+\phi^{\text{opt}}(F_i)A(F_i)\right\}$$
$$-\frac{1}{\sqrt{n}}\sum_{i=1}^{n}\frac{\{R_i-\pi(F_i;\alpha^*)\}}{A(F_i)A_n(F_i)}\{A_n(F_i)-A(F_i)\}$$
$$\times\left\{\hat{B}_n(F_i)-\phi^{\text{opt}}(F_i)A_n(F_i)-B(F_i)+\phi^{\text{opt}}(F_i)A(F_i)\right\}$$

Note that the first term

$$\frac{1}{\sqrt{n}}\sum_{i=1}^{n}\frac{\{R_i-\pi(F_i;\alpha^*)\}}{A(F_i)}\left\{\hat{B}_n(F_i)-\phi^{\text{opt}}(F_i)A_n(F_i)-B(F_i)+\phi^{\text{opt}}(F_i)A(F_i)\right\}$$

can be viewed as the projection of a V-statistic. In particular, $E\left\{\hat{B}_n(F_i)-\phi^{\text{opt}}(F_i)A_n(F_i)\right\}$ converges to $B(F_i)-\phi^{\text{opt}}(F_i)A(F_i)$ as h goes to zero. Up to an $o_p(1)$ term, the above expression can thus be written in the form of Lemma 8.4 in Newey and McFadden (1994) for

$$m_n(z_i,z_j)=\sqrt{n}\frac{\{R_i-\pi(F_i;\alpha^*)\}}{A(F_i)}R_j\left\{d(X_j,Z_j)\epsilon_j(\hat{\beta}_{CCA})-\phi^{\text{opt}}(F_j)\right\}K_h(F_i-F_j)$$
$$m_{n1}(z_i)=\sqrt{n}\frac{\{R_i-\pi(F_i;\alpha^*)\}}{A(F_i)}E\left[R_i\left\{d(X_i,Z)\epsilon_i(\beta^*)-\phi^{\text{opt}}(F_i)\right\}|F_i\right]f_F(F_i)$$
$$m_{n2}(z_i)=0$$
$$\mu=0.$$

By Lemma 8.4 in Newey and McFadden (1994), the above expression is thus

$$O_p\left(E\{||m_n(z_i,z_j)||\}/n+E\left\{||m_n(z_i,z_j)||^2\right\}^{1/2}/n\right)=O_p(n^{-1/2}h^{-r}).$$

This is $o_p(1)$ if $n^{-1/2}h^{-r}$ goes to zero.

Consider now the remainder term

$$||\frac{\{R_i-\pi(F_i;\alpha^*)\}}{A(F_i)A_n(F_i)}\{A_n(F_i)-A(F_i)\}\left\{\hat{B}_n(F_i)-\phi^{\text{opt}}(F_i)A_n(F_i)-B(F_i)+\phi^{\text{opt}}(F_i)A(F_i)\right\}||.$$

When $A(F_i)$ is bounded away from zero, then since $A_n(F_i)$ is uniformly close to $A(F_i)$ because of (E.1) and hence will be bounded away from zero with probability approaching one, the remainder

term is bounded above by

$$C||A_n(F_i) - A(F_i)||||\hat{B}_n(F_i) - \phi^{\text{opt}}(F_i)A_n(F_i) - B(F_i) + \phi^{\text{opt}}(F_i)A(F_i)||$$

for some constant C. The latter term is $O_p\left\{(\log n)(nh^{r+4})^{-1} + h^{2m}\right\}$ and is thus in particular $o_p(1)$ if $(\log n)(nh^{r+4})^{-1}$ and h^{2m} go to zero. It follows by the same reasoning that

$$\frac{1}{4n}\sum_{i=1}^{n}||w(F_i)\left\{\hat{\phi}^{\text{opt}}(F_i) - \phi^{\text{opt}}(F_i)\right\}||$$

is $o_p(1)$.

Assumption 8.1 of Newey and McFadden (1994) includes the condition that the kernel function be zero outside of a bounded set. However, Newey and McFadden suggest that this condition is likely not needed, and indeed we found that a normal kernel function, which does not satisfy the condition, performed better than one that did (Epanechnikov). A normal kernel was thus used in the simulation study and illustrative example.

F. Simulation setup

The following describes the setup used for the simulation study. 1,000 datasets were generated with $n = 1,000$ using the following data generating mechanism. The observation indicator R was generated from a Bernoulli distribution with $P(R = 1) = \pi = 0.5$. We then generated

$$\begin{pmatrix} X \\ Z \\ Y \end{pmatrix} |R \sim N\left\{\begin{pmatrix} \gamma_{X0} + \gamma_X R \\ \gamma_{Z0} + \gamma_Z R \\ \gamma_{Y0} + \gamma_Y R \end{pmatrix}, \begin{pmatrix} \sigma_X^2 & \sigma_{XZ} & \sigma_{XY} \\ \sigma_{XZ} & \sigma_Z^2 & \sigma_{ZY} \\ \sigma_{XY} & \sigma_{ZY} & \sigma_Y^2 \end{pmatrix}\right\}$$

with $\gamma_{X0} = \gamma_{Z0} = \gamma_{Y0} = 0$, $\gamma_X = \gamma_Z = 1$, $\sigma_X^2 = \sigma_Z^2 = \sigma_Y^2 = 1$, and $\sigma_{XZ} = \sigma_{ZY} = \sigma_{XY} = 0.25$. The conditional distribution of R given X, Z and Y is a logistic regression, and via tedious calculations (or Maxima), the coefficient of Y in this logistic regression is shown to be zero if

$$\gamma_Y = \frac{\gamma_X(\sigma_{XY}\sigma_Z^2 - \sigma_{XZ}\sigma_{ZY}) + \gamma_Z(\sigma_{ZY}\sigma_X^2 - \sigma_{XY}\sigma_{XZ})}{\sigma_X^2\sigma_Z^2 - \sigma_{XZ}^2},$$

such that Y and R are conditionally independent given (X, Z), as we assume. It then also follows that $Y|X, Z$ is normal, with expectation $\beta_0 + \beta_X X + \beta_Z Z$, where

$$\beta_X = \frac{\sigma_{XY}\sigma_Z^2 - \sigma_{ZY}\sigma_{XZ}}{\sigma_Z^2(\sigma_X^2 - \frac{\sigma_{XZ}^2}{\sigma_Z^2})}$$

$$\beta_Z = \frac{\sigma_{ZY}\sigma_X^2 - \sigma_{XY}\sigma_{XZ}}{\sigma_X^2(\sigma_Z^2 - \frac{\sigma_{XZ}^2}{\sigma_X^2})}$$

and

$$\beta_0 = \gamma_Y - \beta_X\gamma_X - \beta_Z\gamma_Z$$

In order to calculate $\phi^{\text{opt}}(Y, Z, \beta)$, we require $f(X|Z, Y, R = 1)$, which again is normal, with expectation $\delta_0 + \delta_Z Z + \delta_Y Y$, where

$$\delta_Z = \frac{\sigma_{XY}\sigma_Z^2 - \sigma_{ZX}\sigma_{ZY}}{\sigma_Z^2(\sigma_Y^2 - \frac{\sigma_{YZ}^2}{\sigma_Z^2})}$$

$$\delta_Y = \frac{\sigma_{XZ}\sigma_Y^2 - \sigma_{XY}\sigma_{ZY}}{\sigma_Y^2(\sigma_Z^2 - \frac{\sigma_{ZY}^2}{\sigma_Y^2})}$$

and

$$\delta_0 = \gamma_{X0} + \gamma_X - \delta_Z(\gamma_{Z0} + \gamma_Z) - \delta_Y(\gamma_{Y0} + \gamma_Y)$$

The conditional variance of X given $Y, Z, R = 1$ is given by

$$\sigma_X^2 - (\delta_Z\sigma_{XZ} + \delta_Y\sigma_{XY})$$

G. Bias of an MI MAR analysis

In this appendix we investigate the bias of a standard MI analysis based on the MAR assumption, when in truth missingness in X is affected by X but not outcome Y. We consider the very simple setting in which we assume a continuous outcome Y given a continuous covariate X obeys the following conditional mean model:

$$E(Y|X) = \beta_0 + \beta_X X$$

In order to derive analytical expressions for the bias of an MI analysis assuming MAR, we shall assume the data are generated from the following normal discriminant model

$$R \sim \text{Bernoulli}(\pi)$$
$$\begin{pmatrix} X \\ Y \end{pmatrix} | R \sim N\left\{ \begin{pmatrix} \gamma_{X0} + \gamma_{XR}R \\ \gamma_{Y0} + \gamma_{YR}R \end{pmatrix}, \begin{pmatrix} \sigma_X^2 & \sigma_{XY} \\ \sigma_{XY} & \sigma_Y^2 \end{pmatrix} \right\} \tag{G.1}$$

Standard calculations show that the conditional distribution of R given X and Y is a logistic regression, with corresponding (adjusted) log odds ratios (λ_X, λ_Y) equal to

$$\begin{pmatrix} \lambda_X \\ \lambda_Y \end{pmatrix} = \frac{1}{\sigma_X^2 \sigma_Y^2 - \sigma_{XY}^2} \begin{pmatrix} \sigma_Y^2 \gamma_{XR} - \sigma_{XY}\gamma_{YR} \\ \sigma_X^2 \gamma_{YR} - \sigma_{XY}\gamma_{XR} \end{pmatrix}$$

It follows that Y and R are conditionally independent given X if and only if

$$\gamma_{YR} = \frac{\sigma_{XY}\gamma_{XR}}{\sigma_X^2}$$

which we will assume holds.

As described in the main paper, if $Y \perp\!\!\!\perp R|X$, CCA is valid, due to the fact that $f(Y|X, R = 1) = f(Y|X)$. Under our assumed model and missingness assumption we therefore have $f(Y|X, R) = f(Y|X)$, and so

$$Y|X \sim N\left(\gamma_{Y0} + \frac{\sigma_{XY}}{\sigma_X^2}(X - \gamma_{X0}), \sigma_Y^2 - \frac{\sigma_{XY}^2}{\sigma_X^2} \right)$$

Therefore the parameters (β_0, β_X) of the conditional mean model can be expressed as

$$\beta_0 = \gamma_{Y0} - \frac{\sigma_{XY}\gamma_{X0}}{\sigma_X^2}$$
$$\beta_X = \frac{\sigma_{XY}}{\sigma_X^2}$$

We now derive the values which would be consistently estimated by performing MI (wrongly) assuming MAR. We assume a standard normal linear regression imputation model is used for $X|Y$

$$X|Y \sim N(\delta_0 + \delta_Y Y, \sigma_{X|Y}^2)$$

The imputation model is fitted to the data in the complete cases, in which, from equation (G.1), we have

$$X|Y, R = 1 \sim N\left(\gamma_{X0} + \gamma_{XR} + \frac{\sigma_{XY}}{\sigma_Y^2}\left\{Y - \left(\gamma_{Y0} + \frac{\sigma_{XY}\gamma_{XR}}{\sigma_X^2}\right)\right\}, \sigma_X^2 - \frac{\sigma_{XY}^2}{\sigma_Y^2}\right)$$

and thus the parameters of the imputation model are given by

$$\begin{aligned} \delta_0 &= \gamma_{X0} + \gamma_{XR} - \frac{\sigma_{XY}}{\sigma_Y^2}\left(\gamma_{Y0} + \frac{\sigma_{XY}\gamma_{XR}}{\sigma_X^2}\right) \\ \delta_Y &= \frac{\sigma_{XY}}{\sigma_Y^2} \\ \sigma_{X|Y}^2 &= \sigma_X^2 - \frac{\sigma_{XY}^2}{\sigma_Y^2}. \end{aligned} \tag{G.2}$$

To derive the parameter values consistently estimated by MI assuming MAR it is sufficient to consider the parameters consistently estimated with a single (stochastic) imputation. Thus for subjects with $R = 0$, we (singly) impute their missing X by $X^{imp} = \delta_0 + \delta_Y Y + \epsilon$ where $\epsilon \overset{\text{iid}}{\sim} N(0, \sigma_{X|Y}^2)$. Defining $\tilde{X} = RX + (1 - R)X^{imp}$, let (β_0', β_X') denote the probability limits of the ordinary least squares estimates of the regression of Y on $\tilde{X}$. The slope coefficient of $\tilde{X}$ that is consistently estimated by MI is thus equal to

$$\beta_X' = \frac{\text{Cov}(Y, \tilde{X})}{\text{Var}(\tilde{X})},$$

while the intercept is equal to

$$\beta_0' = E(Y) - \beta_X' E(\tilde{X}). \tag{G.3}$$

We first derive an expression for $\beta'_X = \mathrm{Cov}(Y, \tilde{X})/\mathrm{Var}(\tilde{X})$. The covariance can be expanded as

$$
\begin{aligned}
\mathrm{Cov}(Y, \tilde{X}) &= E\{\mathrm{Cov}(Y, \tilde{X}|R)\} + \mathrm{Cov}\{E(Y|R), E(\tilde{X}|R)\} \\
&= E\left\{R\sigma_{XY} + (1-R)(\delta_Y \sigma_Y^2)\right\} + \mathrm{Cov}\left[\gamma_{Y0} + \gamma_{YR}R, R(\gamma_{X0} + \gamma_{XR}R) + \right. \\
&\quad \left. (1-R)\{\delta_0 + \delta_Y(\gamma_{Y0} + \gamma_{YR}R)\}\right] \\
&= \pi\sigma_{XY} + (1-\pi)\delta_Y\sigma_Y^2 + \gamma_{YR}\mathrm{Cov}\left[R, R(\gamma_{X0} + \gamma_{XR}) + (1-R)\{\delta_0 + \delta_Y\gamma_{Y0}\}\right] \\
&= \pi\sigma_{XY} + (1-\pi)\sigma_{XY} + \gamma_{YR}\mathrm{Cov}\left[R, R(\gamma_{X0} + \gamma_{XR} - \delta_0 - \delta_Y\gamma_{Y0})\right] \\
&= \sigma_{XY} + \pi(1-\pi)\gamma_{YR}(\gamma_{X0} + \gamma_{XR} - \delta_0 - \delta_Y\gamma_{Y0}) \\
&= \sigma_{XY} + \pi(1-\pi)\frac{\sigma_{XY}^3\gamma_{XR}^2}{\sigma_X^4\sigma_Y^2}
\end{aligned}
$$

where we use the fact that $\mathrm{Cov}(R, R) = \mathrm{Var}(R) = \pi(1-\pi)$, and substitute for δ_0 and δ_Y from equations (G.2). The variance of $\tilde{X}$ can be expressed as

$$
\mathrm{Var}(\tilde{X}) = \mathrm{Var}\{E(\tilde{X}|R)\} + E\{\mathrm{Var}(\tilde{X}|R)\}
$$

The variance of the conditional expectation is equal to

$$
\begin{aligned}
\mathrm{Var}\{E(\tilde{X}|R)\} &= \mathrm{Var}\left[R(\gamma_{X0} + \gamma_{XR}R) + (1-R)\{\delta_0 + \delta_Y(\gamma_{Y0} + \gamma_{YR}R)\}\right] \\
&= \mathrm{Var}\left[R(\gamma_{X0} + \gamma_{XR}) + (1-R)\{\delta_0 + \delta_Y\gamma_{Y0}\}\right] \\
&= \mathrm{Var}\{R(\gamma_{X0} + \gamma_{XR} - \delta_0 - \delta_Y\gamma_{Y0})\} \\
&= \pi(1-\pi)\frac{\sigma_{XY}^4\gamma_{XR}^2}{\sigma_X^4\sigma_Y^4}
\end{aligned}
$$

where we use in going from the first to the second line the facts that $R(\gamma_{X0} + \gamma_{XR}R) = R(\gamma_{X0} + \gamma_{XR})$ and $(1-R)\{\delta_0 + \delta_Y(\gamma_{Y0} + \gamma_{YR}R)\} = (1-R)(\delta_0 + \delta_Y\gamma_{Y0})$. The expectation of the conditional

variance is equal to

$$\begin{aligned}
E\{\mathrm{Var}(\tilde{X}|R)\} &= E\left[\mathrm{Var}\left\{RX + (1-R)(\delta_0 + \delta_Y Y + \epsilon)|R\right\}\right] \\
&= E\left\{R^2\sigma_X^2 + (1-R)^2\left(\delta_Y^2\sigma_Y^2 + \sigma_X^2 - \frac{\sigma_{XY}^2}{\sigma_Y^2}\right)\right\} \\
&= E\left\{R\sigma_X^2 + (1-R)\left(\delta_Y^2\sigma_Y^2 + \sigma_X^2 - \frac{\sigma_{XY}^2}{\sigma_Y^2}\right)\right\} \\
&= \sigma_X^2 + E\left\{(1-R)\left(\delta_Y^2\sigma_Y^2 - \frac{\sigma_{XY}^2}{\sigma_Y^2}\right)\right\} \\
&= \sigma_X^2, \text{ since } \delta_Y^2 = \sigma_{XY}/\sigma_Y^2
\end{aligned}$$

and where we use the fact in going from the first to the second line that the variance of the two parts is equal to the sum of their two (conditional variances) because their covariance is zero. Thus we have that

$$\beta_X' = \frac{\sigma_{XY} + \pi(1-\pi)\frac{\sigma_{XY}^3\gamma_{XR}^2}{\sigma_X^4\sigma_Y^2}}{\sigma_X^2 + \pi(1-\pi)\frac{\sigma_{XY}^4\gamma_{XR}^2}{\sigma_X^4\sigma_Y^4}}.$$

The intercept β_0' is equal to

$$\begin{aligned}
\beta_0' &= E(Y) - \beta_X' E(\tilde{X}) \\
&= E\{E(Y|R)\} - \beta_X' E\{E(\tilde{X}|R)\} \\
&= E(\gamma_{Y0} + \gamma_{YR}R) - \beta_X' E\left[R(\gamma_{X0} + \gamma_{XR}R) + (1-R)\{\delta_0 + \delta_Y(\gamma_{Y0} + \gamma_{YR}R)\}\right] \\
&= \gamma_{Y0} + \pi\gamma_{YR} - \beta_X' E\left[R(\gamma_{X0} + \gamma_{XR}) + (1-R)(\delta_0 + \delta_Y\gamma_{Y0})\right] \\
&= \gamma_{Y0} + \pi\gamma_{YR} - \beta_X' E\left[R(\gamma_{X0} + \gamma_{XR}) + (1-R)(\delta_0 + \delta_Y\gamma_{Y0})\right] \\
&= \gamma_{Y0} + \pi\gamma_{YR} \\
&\quad -\beta_X'\left[\pi(\gamma_{X0} + \gamma_{XR}) + (1-\pi)\left\{\gamma_{X0} + \gamma_{XR} - \frac{\sigma_{XY}}{\sigma_Y^2}\left(\gamma_{Y0} + \frac{\sigma_{XY}\gamma_{XR}}{\sigma_X^2}\right) + \frac{\sigma_{XY}}{\sigma_Y^2}\gamma_{Y0}\right\}\right] \\
&= \gamma_{Y0} + \pi\beta_X\gamma_{XR} - \beta_X'\left\{\gamma_{X0} + \gamma_{XR} + (\pi-1)\frac{\sigma_{XY}^2\gamma_{XR}}{\sigma_X^2\sigma_Y^2}\right\}
\end{aligned}$$

The bias in the MAR MI estimate of β_0 is thus equal to

$$\beta_0' - \beta_0 = \pi\beta_X\gamma_{XR} - \beta_X'\left\{\gamma_{X0} + \gamma_{XR} + (\pi-1)\frac{\sigma_{XY}^2\gamma_{XR}}{\sigma_X^2\sigma_Y^2}\right\} + \beta_X\gamma_{X0}.$$

If $\beta'_X \approx \beta_X$, then this expression simplifies to

$$\beta'_0 - \beta_0 = \beta_X(1-\pi)\left(\frac{\sigma^2_{XY}\gamma_{XR}}{\sigma^2_X\sigma^2_Y} - 1\right).$$

To illustrate the magnitude of the biases of an MI analysis assuming MAR, Figure 2 shows, for parameter values $\pi = 0.5$, $\sigma^2_X = \sigma^2_Y = 1$, $\gamma_{X0} = \gamma_{Y0} = 0$, $\gamma_{XR} = 1$, the values of β'_X and β'_0 according to varying values of σ_{XY} (here equal to β_X). The value $\gamma_{XR} = 1$ results in an adjusted log-odds ratio of 1, which would ordinarily be considered a moderately strong missingness mechanism. With these parameter values, from Figure 2 we see that the bias in the slope estimated by an MI analysis assuming MAR is positive but small across all values of β_X. The bias is maximal at a value of $\beta_X \approx 0.76$, but even here the bias is only 5%. The bias in the slope decreases as β_X either decreases to zero or increases to one. The biases of the constant β_0 are larger, and here negative. The bias initially increases as β_X increases, reaching a maximal (absolute) value at $\beta_X \approx 0.61$, before decreasing as β_X approaches one. In view of equation (G.3) and the fact that the bias in β'_X is small, we see that the moderate biases in β'_0 are largely due to the imputed variable $\tilde{X}$ having an incorrect mean, rather than being due to bias in the slope coefficient.

This analysis suggests that, at least in this very simple setting, wrongly assuming MAR when missingness is in fact independent of outcome given the covariate, induces moderately large biases in estimates of the intercept parameter but bias in estimates of the covariate's effect on outcome are modest. However, it is important to note that these expressions were derived under a particular parametric model for the full data and for the missingness mechanism, and that for other data generating mechanisms these conclusions might not hold.

H. Extension to more general patterns of missingness

In this appendix we outline how the proposed approach might be extended to more general patterns of missingness. Suppose that X_1 and X_2 are partially observed, and let the corresponding missingness indicators be R_1 and R_2. Then, assuming that $(R_1, R_2) \perp\!\!\!\perp Y|X_1, X_2, Z$ and that the following models for the missingness probabilities hold:

$$P(R_1 = 1|Y, Z) = P(R_1 = 1|Y, Z; \alpha_1)$$

$$P(R_2 = 1|R_1, R_1X_1, Y, Z) = P(R_2 = 1|R_1, R_1X_1, Y, Z; \alpha_2),$$

an estimator of the target parameter β could be obtained by solving an estimating equation of the form:

$$\begin{aligned} 0 = \sum_{i=1}^{n} & R_{i1}R_{i2}d(X_{i1}, X_{i2}, Z_i)\epsilon_i(\beta) + \{R_{i1} - P(R_{i1} = 1|Y_i, Z_i; \alpha_1)\}\, \phi_1(Y_i, Z_i) \\ & + \{R_{i2} - P(R_{i2} = 1|R_{i1}, R_{i1}X_{i1}, Y_i, Z_i; \alpha_2)\}\, \phi_2(R_{i1}, R_{i1}X_{i1}, Y_i, Z_i), \end{aligned}$$

for arbitrary index functions $d(X_1, X_2, Z), \phi_1(Y, Z)$ and $\phi_2(R_1, R_1X_1, Y, Z)$. Here, the first term corresponds to a standard complete case analysis, the second term allows information to be extracted from the observed data (Y, Z) in subjects with incomplete covariate data, and the third term allows information to be extracted from the observed data (Y, X_1, Z) in subjects with incomplete data in X_2 only. Relative to a standard missing data analysis under MAR, advantages of this proposal are (i) that the missing data assumption $(R_1, R_2) \perp\!\!\!\perp Y|X_1, X_2, Z$ is much easier to interpret than the MAR assumption when the missingness is non-monotone (Robins and Gill (1997)); and (ii) that an MAR analysis using MI requires (correct) specification of the joint covariate distribution $f(X_1, X_2|Y, Z)$, which can be difficult; in contrast, models for $P(R_1 = 1|Y, Z)$ and $P(R_2 = 1|R_1, R_1X_1, Y, Z)$ involve fully observed variables only and tend to be easier to specify. Of course, the efficiency of the estimator would depend on the choices of the functions $d(X_1, X_2, Z), \phi_1(Y, Z)$ and $\phi_2(R_1, R_1X_1, Y, Z)$, and similar steps to those taken in Appendix D.2 could be used to deduce the optimal choices.

References

Daniel, R M, Kenward, M G, Cousens, S N and de Stavola, B L. (2012). Using causal diagrams to guide analysis in missing data problems. *Statistical Methods in Medical Research* **21**, 243–256.

Newey, W K and McFadden, D. (1994). Large sample estimation and hypothesis testing. *In Engle R F and McFadden D L (eds) 'Handbook of Econometrics'*, Elsevier B.V., 2111–2245.

Pearl, J. (1995). Causal diagrams for empirical research. *Biometrika* **82**, 669–688.

Robins, J M. (1997). Non-response models for the analysis of non-monotone non-ignorable missing data. *Statistics in Medicine* **16**, 21–37.

Robins, J M and Gill, R D. (1997). Non-response models for the analysis of non-monotone ignorable missing data. *Statistics in Medicine* **16**, 39–56.

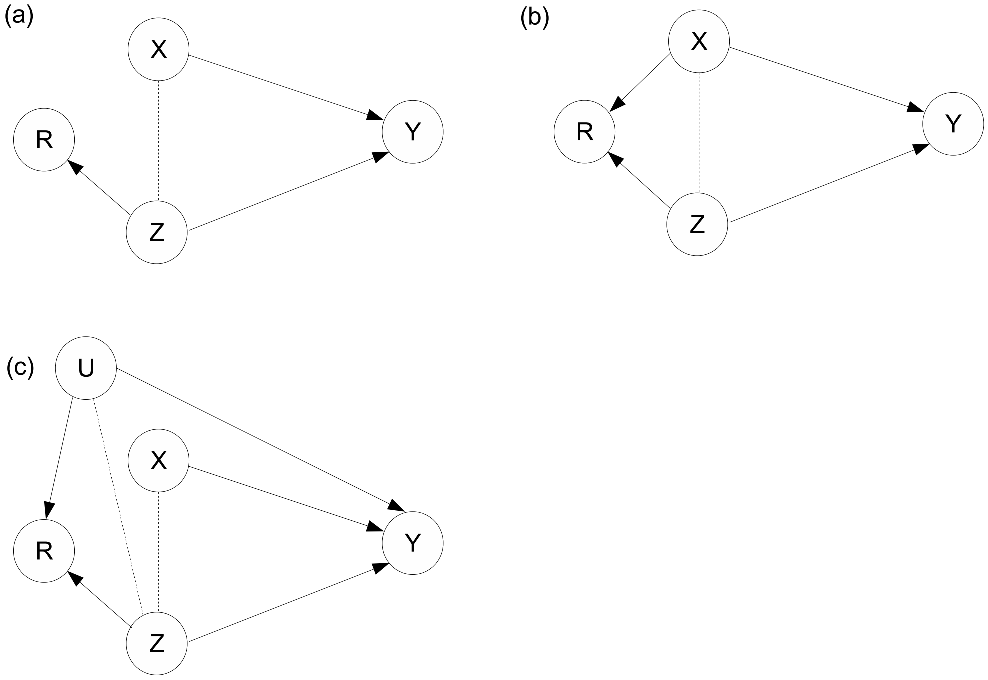

Fig. 1. Causal diagrams depicting various settings which may arise in the context of a prospective cohort study. R denotes whether X is observed or is missing.

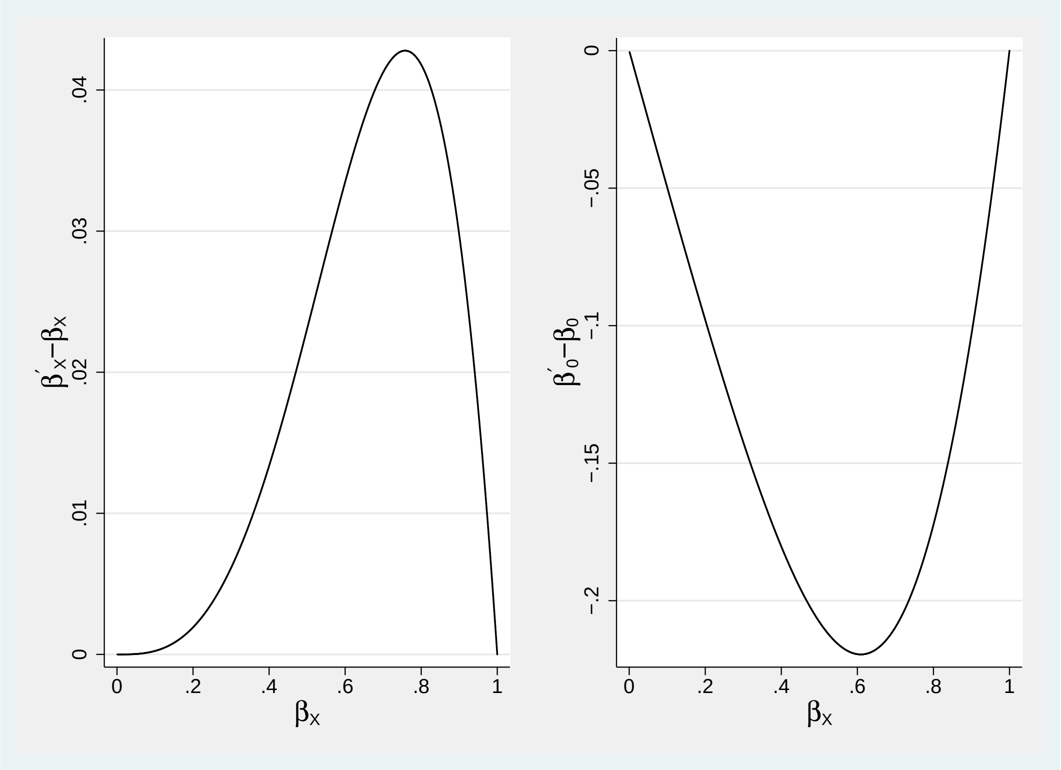

Fig. 2. Bias of multiple imputation assuming MAR: $\beta'_X - \beta_X$ and $\beta'_0 - \beta_0$ as a function of β_X. Here $\beta_0 = 0$. Other assumed parameter values are given in the text
